# Controlled Sonication as a Route to *in-situ* Graphene Flake Size Control

**DOI:** 10.1038/s41598-019-45059-5

**Published:** 2019-06-18

**Authors:** Piers Turner, Mark Hodnett, Robert Dorey, J. David Carey

**Affiliations:** 1 Ultrasound and Underwater Acoustics, National Physical Laboratory, Teddington, Middlesex TW11 0LW United Kingdom; 2 Centre for Engineering Materials, Department of Mechanical Engineering Sciences, University of Surrey, Guildford, Surrey GU2 7XH United Kingdom; 3 Advanced Technology Institute, University of Surrey, Guildford, Surrey GU2 7XH United Kingdom; 4 Department of Electrical and Electronic Engineering, University of Surrey, Guildford, Surrey GU2 7XH United Kingdom

**Keywords:** Synthesis and processing, Synthesis of graphene

## Abstract

Ultrasonication is widely used to exfoliate two dimensional (2D) van der Waals layered materials such as graphene. Its fundamental mechanism, inertial cavitation, is poorly understood and often ignored in ultrasonication strategies resulting in low exfoliation rates, low material yields and wide flake size distributions, making the graphene dispersions produced by ultrasonication less economically viable. Here we report that few-layer graphene yields of up to 18% in three hours can be achieved by optimising inertial cavitation dose during ultrasonication. We demonstrate that inertial cavitation preferentially exfoliates larger flakes and that the graphene exfoliation rate and flake dimensions are strongly correlated with, and therefore can be controlled by, inertial cavitation dose. Furthermore, inertial cavitation is shown to preferentially exfoliate larger graphene flakes which causes the exfoliation rate to decrease as a function of sonication time. This study demonstrates that measurement and control of inertial cavitation is critical in optimising the high yield sonication-assisted aqueous liquid phase exfoliation of size-selected nanomaterials. Future development of this method should lead to the development of high volume flow cell production of 2D van der Waals layered nanomaterials.

## Introduction

Since the discovery of graphene^1^ and the characterisation of its properties^1–3^, it has shown huge potential in applications ranging from energy storage^4^, solar cells^5^, printed electronics^6^, composite fillers^7^, and water filtration membranes^8^. The discovery of graphene has also generated significant research interest into other 2D van der Waals layered nanomaterials, such as the family of metallic and semiconducting transition metal dichalcogenides^9^. One of the main challenges limiting the further applications and commercialisation of graphene and other 2D layered materials is that it remains difficult to produce large quantities of high-quality flakes with application specific size distributions. Many of the useful properties of graphene are indeed dependent on the lateral size and thickness of individual flakes^3,5^; for example, graphene flakes with large lateral dimensions (>1 *μ*m) are used in polymer composites^10^ and conductive graphene inks^6^, flakes with smaller lateral dimensions (<1 *μ*m) are employed in ceramic composites^11^, and graphene quantum dots (<100 nm) are found in photovoltaics, fuel cells, and catalysis applications^12^.

One of the most scalable graphene production routes is to exfoliate it from graphite in the liquid phase using ultrasonication^13,14^, shear mixing^15^, wet-jet milling^16^, or microfluidisation^17^. Although some liquid phase exfoliation methods report graphene yields as high as 100%^16,17^, a recent study demonstrated that the majority of commercially available graphene had less than 10% graphene content. This was due to the flakes containing more than 10 layers, which makes them nano graphite rather than multilayer graphene according to the ISO standard on graphene nomenclature^18^. Due to the wide flake size distributions (nm-*μ*m) that are characteristic of liquid phase exfoliation techniques extensive centrifugation is often required to both narrow the size distribution and remove large graphitic flakes. Although cascade centrifugation has been shown to be effective in isolating narrow size distributions, it is time intensive, lowers the bulk concentration of the dispersed graphene and can inadvertently remove thinner graphene flakes with larger lateral dimensions as it separates flakes by their length^19^. As liquid phase exfoliation techniques typically produce dispersions with low intrinsic graphene concentrations (~0.1 mg ml^−1^), centrifugation and re-dispersion is often required to produce graphene dispersions with industrially viable concentrations (≥1 mg ml^−1^). As such, the removal of large graphene flakes can be unavoidable when producing graphene dispersions.

Despite microfluidisation, shear mixing and wet jet milling demonstrating superior exfoliation rates of pristine graphene flakes^15–17^, ultrasonication is one of the most widely used standalone methods to produce high-quality graphene dispersions, as well as being a complementary processing technique in other graphene production methods. The main limitations of using ultrasonication are its wide flake size distributions, low exfoliation rates, and comparatively low graphene yields. This is due to a poor understanding of the fundamental mechanisms driving graphene exfoliation, and a reliance upon purely empirical parameters such as sonication time^13^, temperature control^20^ and nominal electrical input power^21^ to monitor and develop ultrasonication strategies. Although acoustic cavitation is often suggested as the primary mechanism driving graphene exfoliation during sonication^22,23^, this has not been experimentally validated, and there have been no efforts to directly control and optimise acoustic cavitation during sonication. Using advanced cavitation metrology tools, we demonstrate that inertial cavitation drives graphene exfoliation during sonication for the first time. Furthermore, a controlled application of inertial cavitation is critical for optimising the sonication-assisted liquid phase exfoliation of graphene, resulting in improved graphene exfoliation rates, as well as a route towards *in-situ* size control of the graphene flakes. These findings will be instrumental in developing advanced ultrasonication strategies that will increase the large volume production and commercialisation of a wide range of 2D nanomaterials.

## Acoustic Cavitation

**Figure 1:**
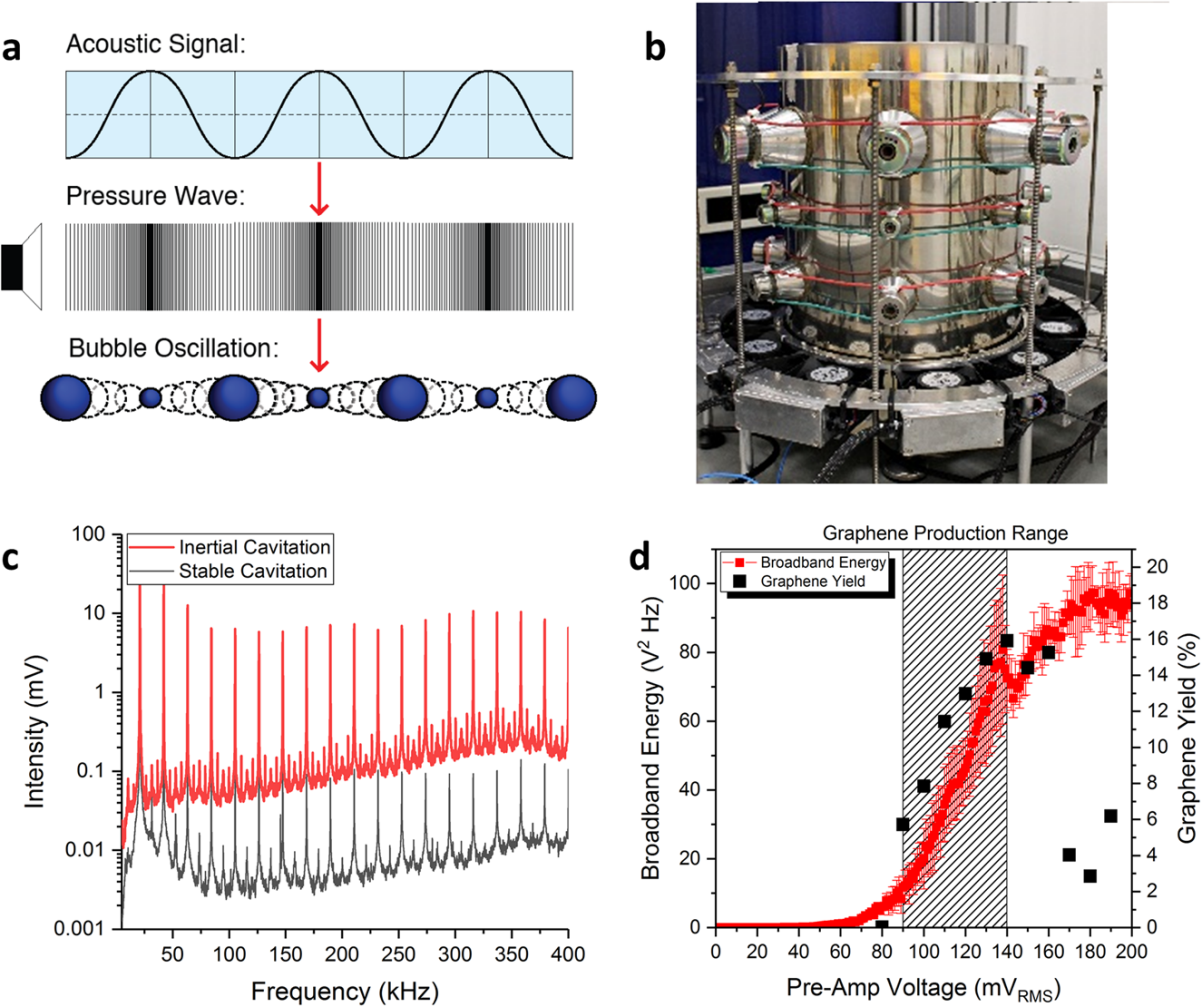
(**a**) Schematic of the growth and collapse of cavitating bubbles in response to an applied acoustic field and the associated pressure wave. (**b**) Photograph of the National Physical Laboratory’s 17-litre multi-frequency reference cavitating vessel with transducers around the circumference. (**c**) The frequency spectrum of cavitation signals arising from stable and inertial cavitation. The presence of harmonic activity is indicative of stable cavitation activity, and the rise in the background noise is indicative of inertial cavitation activity. (**d**) E_cav_ and the graphene yield as a function of the pre-amp voltage (the output voltage of a signal generator that was used to drive the reference vessels top row of 21.06 kHz transducers via a 400 W power amplifier). The hashed rectangle represents the pre-amp voltage range over which graphene was produced in this study. The uncertainty in E_cav_ is associated with the standard deviation of five independent measurements.

Acoustic cavitation is the stimulated expansion and collapse of microbubbles in response to an applied acoustic field (Fig. 1a). Sonic baths and sonic horns generate acoustic cavitation by exciting a fluid with continuous or pulsed pressure waves at kHz frequencies. A regime with cavitating bubbles that have long lifetimes is referred to as non-inertial or stable cavitation, whereas inertial cavitation is characterized by short-lived cavitating bubbles which undergo violent and chaotic collapse^24^. Both types of cavitation exhibit physicochemical effects which are strongly dependent on the properties of the liquid being sonicated (density, viscosity and boiling point) as well as the acoustic field frequency, amplitude and geometry^25^. Stable cavitation generates short range vortices known as microstreaming, whereas inertial cavitation collapses radiate spherical shockwaves with velocities of up to 4,000 m s^−1^ with peak pressures of up to 6 GPa^26^. Intense liquid jets (jetting) with pressures of up to 1 GPa can also be generated during inertial collapse^27^. In a typical sonication environment, such as a sonic bath or sonotrode, both types of cavitation can exist simultaneously.

In this work, acoustic cavitation was generated by a multi-frequency reference cavitating vessel (Fig. 1b) that can produce stable and reproducible cavitation fields with a well-defined acoustic field distribution^28^. The acoustic signals that cavitating bubbles emit (Fig. 1c) were measured using a calibrated needle hydrophone. Inertial cavitation was delineated from stable cavitation by quantifying the broadband noise, over a MHz frequency range in which harmonic activity is indistinguishable from the background noise^29^. This was carried out by measuring the high frequency broadband energy^30^ (Equation 1), parametrised as ${E}_{cav}$.

1 $${E}_{cav}=\frac{1}{N}{\sum }_{t=1}^{N}{\int }_{{f}_{1}}^{{f}_{2}}{V}_{c}{(f)}^{2}df$$

where ${V}_{c}(f)$ are the spectral magnitudes measured from the frequency domain cavitation spectra (Fig. 1c) and *f*_1_ and *f*_2_ are 1.5 MHz and 2.5 MHz, respectively. The inertial cavitation threshold was determined by measuring ${E}_{cav}$ as a function of the nominal input power of the vessel; the measurement protocol is described in the Supplementary Information 1. As shown in Fig. 1d, the inertial cavitation threshold is characterised by a systematic rise in ${E}_{cav}$^29^. This occurs above a pre-amp voltage of ~60 mV_RMS_, which corresponds to a nominal input electrical power of 5 Watts (corresponding to a vessel power density of around 0.3 W L^−1^).

To study graphene exfoliation arising from the physiochemical effects of acoustic cavitation, samples were produced by sonicating graphite in 28 ml low density polyethylene vials positioned in a region where inertial cavitation activity is intense and localised (Fig. S1.2a). Preliminary experiments found that graphene is first produced only after the onset of the inertial cavitation (Fig. 1d), which demonstrates that the physiochemical effects of inertial cavitation drive graphene exfoliation during ultrasonication. At high pre-amp voltages (high acoustic powers) ${E}_{cav}$ saturates due to cavitation shielding^31^, where a significant volume fraction of cavitating bubbles dynamically scatter and absorb the acoustic field. As there is a significant drop in the graphene exfoliation rate at high pre-amp voltages, Fig. 1d also suggests that cavitation shielding adversely affects the graphene exfoliation rate, however more work will need to be carried out to experimentally validate this. The highly non-linear nature of inertial cavitation combined with the significant perturbation of the graphene exfoliation rate at high acoustic powers (Fig. 1d) suggests that measurement and control of inertial cavitation is essential when developing sonication methodologies.

## Results and Discussion

To explore the role of inertial cavitation on the liquid phase exfoliation of graphene, graphite with a narrow 45–75 *μ*m size distribution (Supplementary Information 2.3), was exfoliated over a range of both pre-amp voltages (shown in Fig. 1c) and sonication times. A low initial graphite concentration (0.2 mg ml^−1^) was chosen to increase the dispersed graphene yield and minimise the population of the larger graphitic flakes post sonication, ensuring that the graphene size distributions are representative of the acoustic cavitation mechanisms which exfoliated them. As ${E}_{cav}$ is a direct and real time measurement of the inertial cavitation activity, which is the stimulus driving the liquid phase exfoliation of graphene, multiplying ${E}_{cav}$ by the total sonication time, t, quantifies the accumulated dose of inertial cavitation (ICD) experienced by the graphite and graphene flakes during sonication. This method for characterising the accumulated inertial cavitation activity has also featured in medical ultrasound studies employing analogous measurement protocols^32–34^. As the value of $\,{E}_{cav}$, and therefore ICD is dependent on the waveform capture settings (vertical resolution, timebase and sampling rate), the MHz frequency band over which it is calculated, the frequency response of the hydrophone, and the hardware filtering and amplification in the signal chain (Fig. S1.1), absolute values obtained are arbitrary, though the units of ICD can be considered as volts squared. However, as the ${E}_{cav}$ measurements in this work were carried out using the same measurement protocol, the resultant ICD measurements are directly comparable.

**Figure 2:**
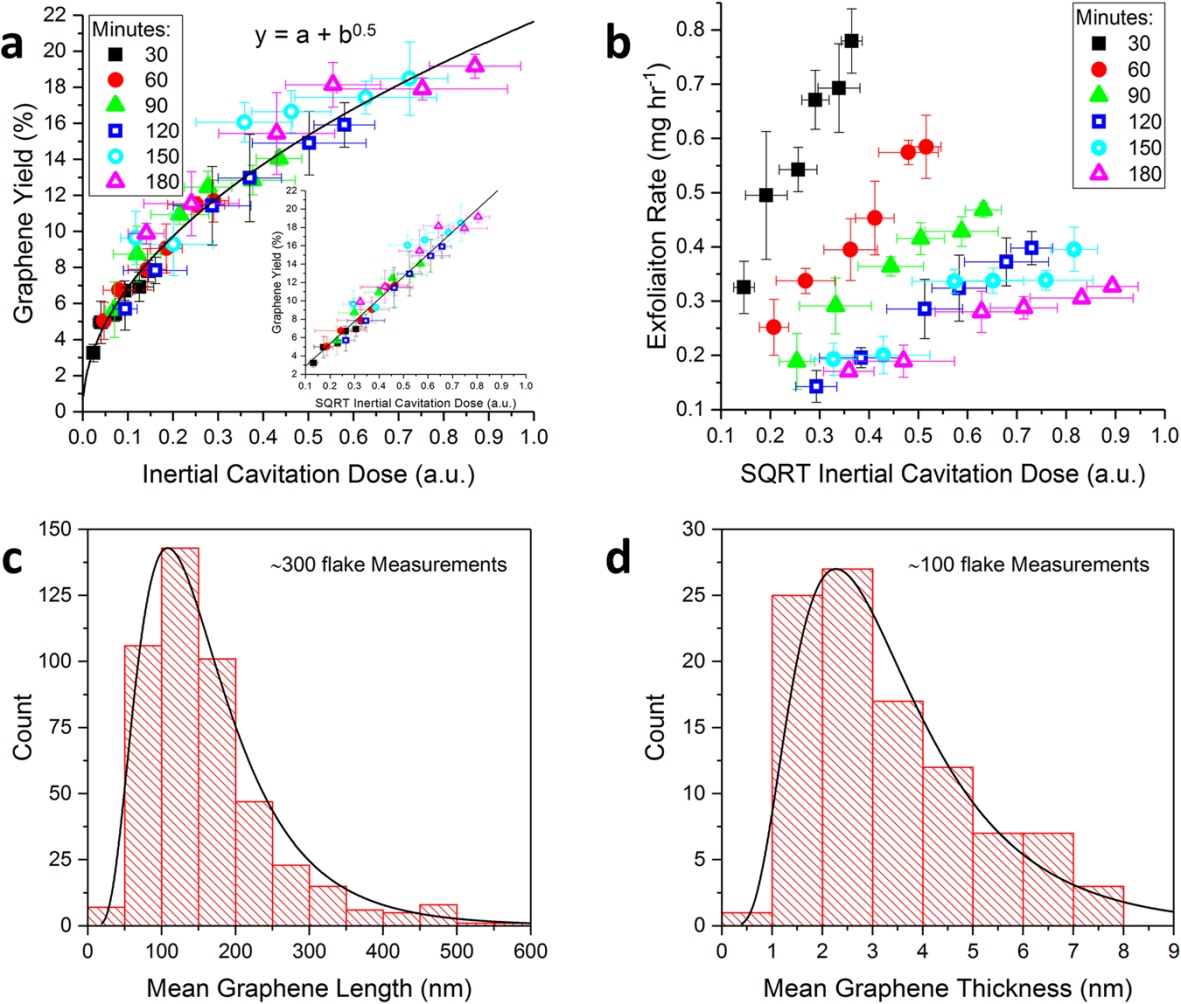
(**a**) The graphene yield (after centrifugation) as a function of the ICD and (inset) the square root of the ICD. (**b**) The graphene exfoliation rate as a function of the square root of the ICD. The symbols in (**a**) and (**b**) delineate the data as a function of sonication time, measured in minutes. Representative log-normal plots of (**c**) graphene length and (**d**) thickness distributions, measured from a dispersion which was sonicated for 150 minutes at a pre-amp voltage of 140 mV_RMS_, using SEM and AFM respectively. The uncertainty in the graphene yield is associated with the standard deviation of three graphene yield measurements at each sonication time and the uncertainty in ICD is associated with the standard deviation of five independent high frequency broadband energy measurements.

Figure 2a shows that the graphene yield exhibits a power law relationship with ICD, such that there is a linear relationship between the graphene yield and the square root of the ICD (Fig. 2a, inset). As the ICD is a product of $\,{E}_{cav}$ and sonication time, this square root relationship explains the observation of the graphene yield increasing as a function of the square root of sonication time^13^. As such, the graphene exfoliation rate can be increased for a given sonication time by increasing the intensity of the inertial cavitation activity, as measured by the square root of ICD (Fig. 2b). However due to cavitation shielding affects the inertial cavitation activity cannot be indefinitely increased by increasing the input power (Fig. 1d). Although the highest graphene yield (~18%) was amongst the highest in the sonication literature, the low initial graphite concentration results in an exfoliation rate which is less optimal for volume production. As such, Fig. 2a demonstrates inertial cavitation activity drives graphene exfoliation during sonication, and the graphene exfoliation rate is dependent on inertial cavitation dose rather than alternative cavitation metrics (Supplementary Information 3).

During exfoliation trials, it was found that sonication times beyond 120 minutes resulted in anomalously high graphene yields that appeared to deviate from the power law relationship with ICD (Fig. S2.5a). This was subsequently found to occur due to the temperature of the water in the LDPE vials increasing during long periods of sonication (Fig. S2.5b), resulting in faster bubble growth rates and therefore a greater frequency of inertial cavitation collapses^35^. Stable temperatures were maintained over long (150 and 180 minutes) sonication times by using an array of cooling fans to actively cool the vessel. As such, cooling strategies should be carefully considered when developing large volume sonication methodologies to ensure consistent exfoliation. Shorter sonication times, as well as pulsed ultrasonication, may help mitigate temperature increase during sonication.

**Figure 3:**
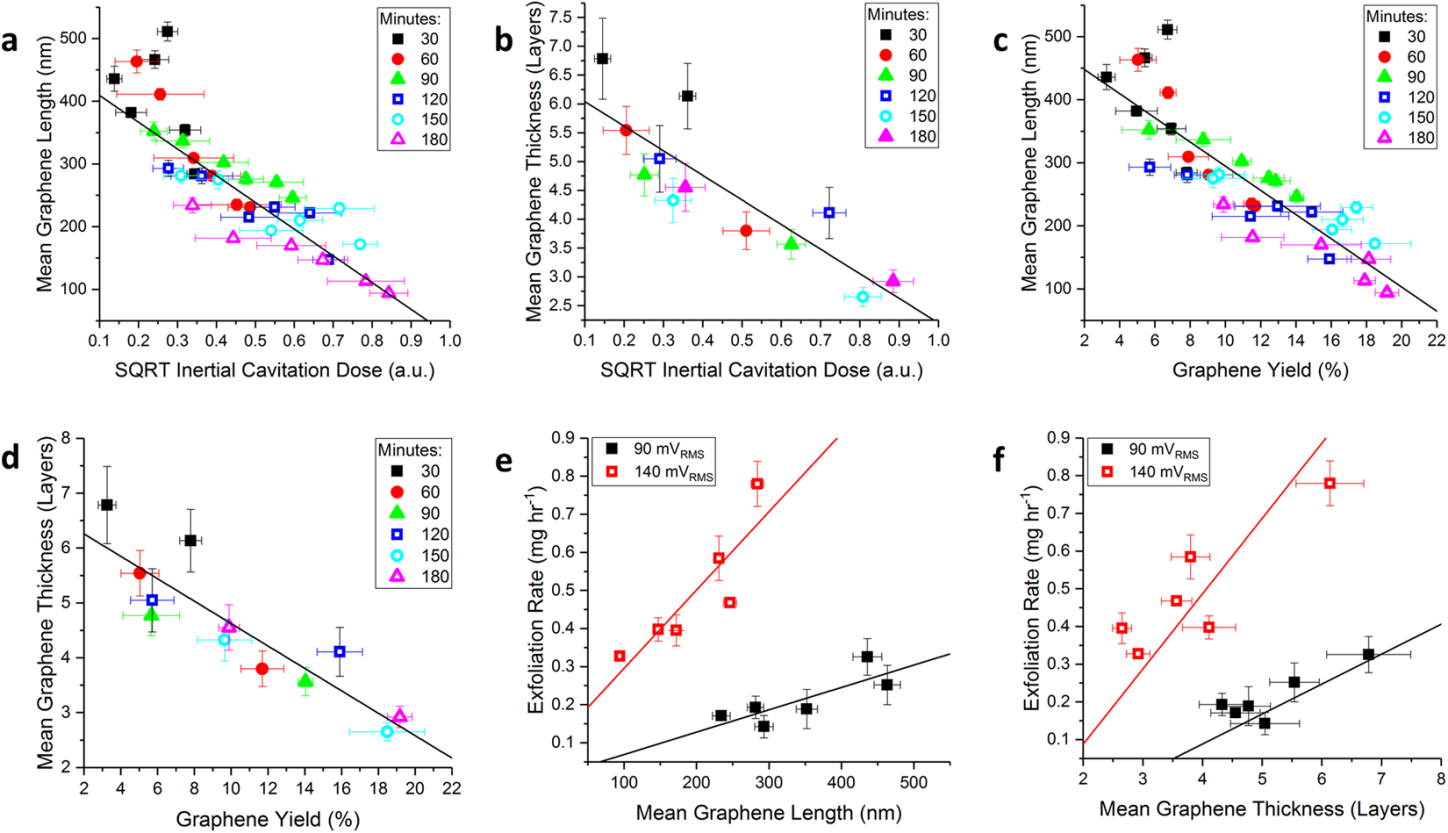
The mean graphene (**a**) length and (**b**) thickness as a function of the ICD. The mean graphene (**c**) length and (**d**) thickness as a function of the graphene yield. The symbols in (**a**–**d**) delineate the data as a function of sonication time, measured in minutes. The graphene exfoliation rate (${c}_{g}\,{t}^{-1}$) as function of (**e**) the mean graphene length and (**f**) thickness for graphene samples produced with the highest and lowest pre-amp voltages (acoustic powers) used in this work. The uncertainty in the graphene yield and exfoliation rate is associated with the standard deviation of three graphene yield measurements at each sonication time, and the uncertainty in graphene length and thickness is associated with the standard error of ~300 and ~100 measurements, respectively.

To quantitatively investigate the evolution of the graphene size distribution as a function of ICD, the length and thickness of the graphene flakes were measured using scanning electron microscopy (SEM) and atomic force microscopy (AFM). The graphene length (Fig. 2c) and thickness (Fig. 2d) distributions were log-normal in shape, implying a multiplicative stochastic fracturing mechanism, whereas a bimodal distribution would be indicative of an erosion process^36^. Accordingly, it can be concluded that inertial cavitation, which is characterised by stochastic and energetic bubble collapse, fractures graphite/graphene during sonication in a multiplicative stochastic process. Figure 3a,b show that the mean length and thickness of graphene flakes decreases linearly as a function of the square root of the ICD. As the <0.5 *μ*m lateral dimensions of the flakes in the post-sonication precipitate (Fig. S2.4) are significantly smaller than the dimensions of the initial graphite population (45–75 *μ*m), this indicates that the entire initial graphite population has been fractured by the accumulated inertial cavitation activity during sonication. Therefore, during sonication the mean flake size will progressively decrease until the flakes are small enough to be suspended in the solution by the electrostatic repulsion of the adsorbed sodium cholate surfactant molecules. This is demonstrated in Fig. 3c,d, which show that the mean graphene length and thickness are both linearly correlated (Pearson’s R ~0.9) with the graphene yield.

The linear relationships between the graphene exfoliation rate and the flake size measurements in Fig. 3e,f indicate that the physical size of the graphite/graphene flakes limits the rate at which it is exfoliated during ultrasonication. This finding demonstrates that inertial cavitation preferentially exfoliates larger flakes during ultrasonication. Such a size preference likely arises from the increased size and/or surface area of larger flakes, which absorb a greater fraction of the shockwave energy generated by nearby inertially cavitating bubbles. Larger graphite flakes will also have an increased probability of containing structural defects such as holes or tears, resulting in a greater fracturing potential. Conversely, there may be a critical size below which graphite cannot be further exfoliated by continued sonication. This has been observed for graphene oxide^37^ and carbon nanotubes^38^ and is demonstrated by the saturation in the graphene yield as a function of ICD despite there being an abundance of ~0.5 *μ*m size graphite flakes in the precipitate (Fig. S2.4). As such, inertial cavitation preferentially exfoliates larger ~0.5 *μ*m size graphite flakes, whereas small dispersed flakes are indirectly exfoliated by the physiochemical effects of nearby inertial cavitation activity.

Graphene exfoliation is likely to be driven by a combination jetting, microstreaming and shockwaves during sonication. As jetting is facilitated by nearby extended surfaces, and the maximum size of the graphite used (sieved to 45–75 *μ*m) is much smaller than the resonant size of the cavitating bubbles present in this work (~160 *μ*m at 21.06 kHz), jetting events within the graphene dispersion will significantly decrease in frequency as the mean flake size decreases during sonication. However, jetting may also exfoliate any graphite/graphene flakes in the vicinity of the LDPE vials’ walls. The local shear stresses generated by collapsing bubbles, known as microstreaming, are also unlikely to drive exfoliation as graphene was not observed below the inertial cavitation threshold where microstreaming still occurs. Furthermore, as the speed of microstreaming vortices is proportional to the squared frequency of cavitating bubbles^39^, the shear forces they generate will be more effective at megasonic frequencies^40^. Consequently, shockwave exfoliation, which is facilitated by a combination of fracturing^41^ and the high velocity interparticle collisions^42,43^, is the most probable exfoliation mechanism during sonication. However, as shockwaves lose more than 50% of their initial energy over the first 25 *μ*m of propagation due to absorption^26^, and will be attenuated by dispersed graphene flakes (which will increase in density during sonication), graphene exfoliation is most likely facilitated by the shockwaves generated by immediately adjacent inertial cavitation collapse events.

### Raman characterisation for material quality and flake size quantification

**Figure 4:**
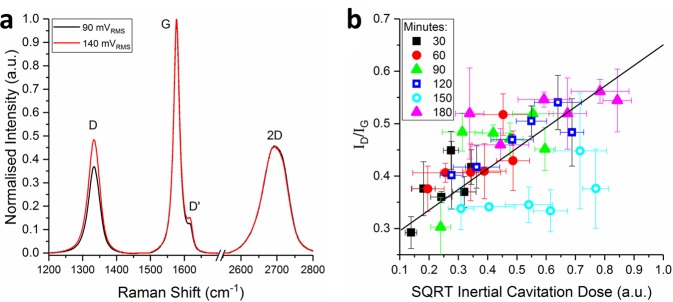
(**a**) Normalised Raman spectra of graphene dispersions produced at the maxima and minima of the ICD range. (**b**) The ID/IG ratio as a function of the square root of the ICD. The uncertainty in the ID/IG ratio and the ICD is associated with the standard deviation of three Raman measurements and five broadband energy measurements per sample, respectively.

Spectroscopic trends in the graphene samples were explored using visible Raman spectroscopy. To ensure that the measurements were representative of the graphene dispersions, 120 spectra were collected and averaged across 20 × 20 *μ*m areas of re-stacked graphene films. These films, which were produced using vacuum filtration, contain dense ordered networks (~50% free volume) of nanosheets such that the laser beam (532 nm excitation wavelength) interrogates 100 s of flakes per measurement^44^. The Raman spectrum of graphene, shown in Fig. 4a, consists of the characteristic G, D, and 2D peaks, which are indicative of many properties including functionalisation, strain, defects, and size distribution^44–48^. Specifically it has been established that the ratio of the intensity of the D peak (~1320 cm^−1^) to that of the G peak (~1580 cm^−1^) is indicative of graphene nanosheet size, and the shape and intensity of the 2D peak (~2700 cm^−1^) changes as function of graphene nanosheet thickness^44,47^.

Figure 4a shows that the ratio of the G peak to the D peak intensity, I_D_/I_G_, increases between the minima and maxima of the ICD range. As the inverse of the I_D_/I_G_ ratio is proportional to the length of graphene flakes^44^, Fig. 4b shows that the graphene length is decreasing over the ICD range. Although this trend mirrors the quantitative SEM length distributions in Fig. 3a, it is less strongly correlated due to the intentionally low centrifugation speeds used in this work (1000 rpm, 120 g) which were chosen to minimise the effects of centrifugation on the dispersed graphene population. The resulting wide size distributions are evidenced by the large variances in the I_D_/I_G_ ratio that were observed across each two-dimensional Raman map (Fig. S4.2a). This is likely the cause of the large uncertainties and anomalies in the I_D_/I_G_ ratio (Fig. 4b) measured from graphene samples produced at a high ICD.

The I_2D_/I_G_ intensity ratio was ~0.45 across the entire ICD range, suggesting a mean graphene thickness of ~5 layers^44^, whereas the AFM data suggested a decreasing thickness as a function of ICD (Fig. 3b). This inconsistency is likely due to the large variances in the I_2D_/I_G_ ratio that were observed across each two-dimensional Raman map (Fig. S4.2b) and suggests that is challenging to get representative Raman data from restacked graphene films containing wide flake size distributions. Alongside graphene size distribution analysis, Raman spectroscopy can also be used to quantify the defects in graphene. Eckmann *et al*.^48^ reported that the ratio of the D peak to the D’ peak was dependant on the defect type, such that an I_D_/I_D’_ ratio of ~13, ~7, and ~3 indicates the presence of sp^3^ defects, vacancy defects and, edge defects respectively. As I_D_/I_D’_ was found to be ~3 for all graphene samples in this work, this shows that inertial cavitation causes flake scission without introducing a significant number of basal plane defects in graphene during sonication.

## Conclusion

In conclusion, by optimising the inertial cavitation dose higher graphene exfoliation rates can be achieved over shorter sonication times, with minimal temperature increases and low nominal input powers. We demonstrate that inertial cavitation preferentially exfoliates larger flakes and that the graphene exfoliation rate and flake dimensions are strongly correlated with, and therefore can be controlled by, inertial cavitation dose; which is a direct measurement of the violent collapses that are indicative of inertial cavitation. During sonication graphite is fractured in a multiplicative process by the shockwaves generated by immediately adjacent inertial cavitation activity; few basal plane defects are incorporated. We also show that temperature increase during sonication can result in inconsistent exfoliation, and therefore temperature control strategies should be employed to ensure consistent ultrasonication. More generally, we show that careful measurement and control of acoustic cavitation is critical when developing efficient ultrasonication methodologies and which by extension can ultimately lead to high volume flow cell production of 2D van der Waals layered nanomaterials.

## Methods

Pre-treated and sieved Asbury Carbons fine flake graphite (0.2 mg ml^−1^) was sonicated in LDPE Nalgene vials (28 ml, Fisher Scientific) with sodium cholate surfactant (3 mg ml^−1^, Sigma Aldrich). The LDPE vials were sonicated continuously at 21.06 kHz for up to 3 hours in NPL’s multi-frequency reference vessel. After sonication, the graphene dispersions were left to sediment overnight before being centrifuged at 1000 rpm (120 rcf) for 2 hours before being characterised with UV-Vis, AFM, SEM and Raman spectroscopy. The graphene yield is given by $(({c}_{g}/{c}_{gi})\ast 100)$, where ${c}_{g}$ is the graphene concentration that is calculated from UV-Vis spectra, and ${c}_{gi}$ is the concentration of the pre-treated and sieved graphite. Acoustic cavitation measurement details, experimental methodology development, and the graphene characterisation methods are described in detail in the supplementary information.

## Supplementary information

Supplementary Information: Controlled Sonication as a Route to in-situ Graphene Flake Size Control

## Data Availability

Details of the data and how to request access are available from the University of Surrey Publications Repository.
