# Supplementary material for: Controlled Sonication as a Route to in-situ Graphene Flake Size Control
Source: Sci Rep. 2019 Jun 18;9:8710. doi: 10.1038/s41598-019-45059-5 (PMC6581953; doi:10.1038/s41598-019-45059-5)
Supplement: Supplementary file 1 — Supplementary Information: Controlled Sonication as a Route to in-situ Graphene Flake Size Control [file 41598_2019_45059_MOESM1_ESM.pdf]

Supplementary Information:

Controlled Sonication as a Route to in-situ Graphene Flake Size Control

Piers Turner[1*], Mark Hodnett[1], Robert Dorey[2] and J. David Carey[3,4*]

[1]Ultrasound and Underwater Acoustics, National Physical Laboratory, Teddington, Middlesex, TW11 0LW, United Kingdom.

[2]Centre for Engineering Materials, Department of Mechanical Engineering Sciences, University of Surrey, Guildford, Surrey, GU2 7XH, United Kingdom.

[3]Advanced Technology Institute, University of Surrey, Guildford, Surrey, GU2 7XH, United Kingdom.

[4]Department of Electrical and Electronic Engineering, University of Surrey, Guildford, Surrey, GU2 7XH, United Kingdom.

Corresponding authors

*P.T. email piers.turner@npl.co.uk

*J.D.C. email david.carey@surrey.ac.uk

Contents

S1 Acoustic cavitation measurements 3

S2 Graphene production methodology 6

S2.1 Choice of vial 6

S2.2 Wetting of the vials 7

S2.3 Choice of Graphite and Surfactant 8

S2.4 Cooling strategy 11

S3 Alternative acoustic cavitation metrics 12

S4 Characterisation Methods 16

S4.1 UV-Vis Spectroscopy 16

S4.2 Raman Spectroscopy 17

S4.3 Atomic force microscopy 20

S4.4 Scanning Electron Microscopy 22

References 25

S1 Acoustic cavitation measurements

Acoustic cavitation measurements were carried out using an Onda HCT 0310 Needle hydrophone. An NPL Cavimeter[1] was used for hardware filtering and amplification of a low frequency (kHz) channel and high frequency (MHz) channel. The low frequency, (LF), and high frequency, (HF), channels were interrogated using a two channel Picoscope 5242B USB oscilloscope (Figure S1.1). The Picoscope measured the time domain cavitation signals from the LF and HF channels simultaneously using a 15-bit vertical resolution and 0.1 kHz frequency resolution. The HF broadband energy, and therefore the inertial cavitation dose, was calculated over a frequency range (1.5-2.5 MHz) where a significant increase in broadband noise was detected as a function of pre-amp voltage (Figure 1.1b). LabVIEW software was written to both drive the multi-frequency reference vessel, via the output channel of the Picoscope, and process live cavitation signals measured by the Picoscope. Cavitation measurements were made by pulsing the signal generator output of the Picoscope. The pulse duration was four seconds and the dwell time was eight seconds. The pulsed mode operation minimised temperature build up and allowed large bubbles to dissipate between measurements such that cavitation hysteresis was mitigated.

The ~40 waveforms collected during each four second measurement were fast Fourier transformed and averaged before a full range of spectral measurements were performed. To ensure the acoustic cavitation measurements were representative of the cavitation fields that the graphene samples would be subjected to, the acoustic field measurements were performed with the HCT needle hydrophone positioned within an LDPE vial. The LDPE vials were filled with, and were pre-soaked in, the same water as the vessel; the reference vessel was refilled between measurements with de-ionised and filtered water that was mixed with 0.2% by volume of MICRO-90 Cole Palmer surfactant.

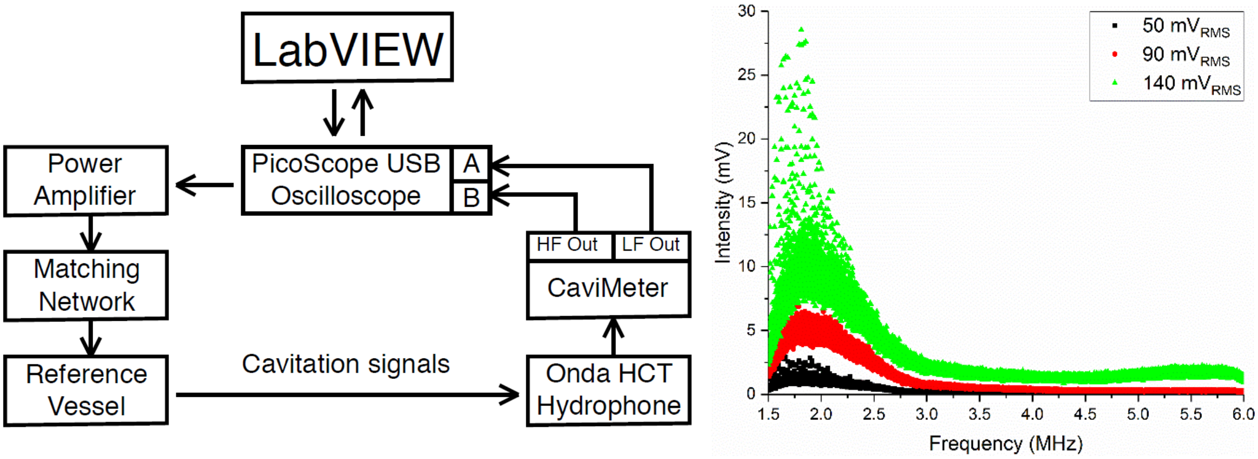

Figure S1.1: *(Left) Schematic of the drive electronics used to drive the multi-frequency reference vessel. (Right) The frequency domain cavitation spectra from the HF channel of the Picoscope at three different pre-amp voltages, showing that the broad band noise between 1.5-2.5 MHz undergoes the most significant change as the pre-amp voltage in increased.*

To ensure more repeatable sonication, an off-axis pressure peak (Figure S1.2a) was used to locate vials within during exfoliation. This location was chosen as it had a minimal temperature increase as a function of pre-amp voltage (Figure S1.2b) and had a more consistent and repeatable acoustic field (Figure S1.2c, S1.2d). Conversely, the on-axis pressure peak is subject to increased temperatures due to the superposition of the acoustic field from the cylindrical arrays of transducers, resulting in higher pressures (Figure S1c) and therefore increased inertial cavitation activity (Figure S1.2d). As such, all graphene work was carried in this off-axis peak location.

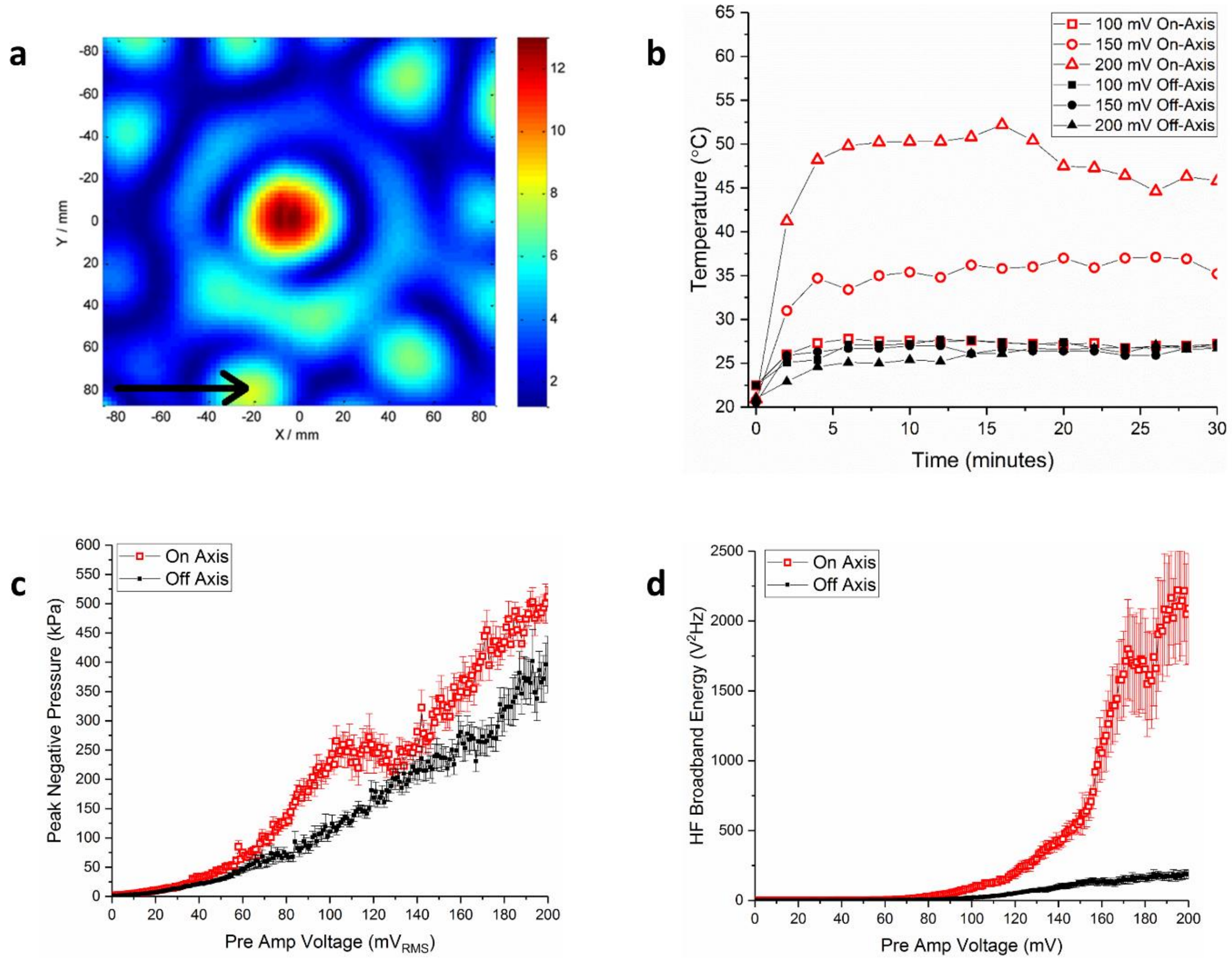

Figure S1.2: *Acoustic field measurements. (a) A peak negative pressure map of the multi-frequency reference vessel driven by its top row of 21.06 kHz transducers (adapted from Wang et al. [2]), with an inset arrow marking the location of the off-axis peak that was used for the majority of sonication experiments in this study. The peak negative pressure is a measure of the rarefactional tensile stress that the water in the vessel is being subjected to, the higher peak negative pressure the higher the rarefactional stress the larger the volume pulsations that cavitating bubbles undergo. (b) The temperature increase within a 15 ml polypropylene (PP) vial located within both off- and on-axis peaks as a function of pre-amp voltage and sonication time. (c) The peak negative pressure and (d) the HF broadband energy as a function of the pre-amp voltage within PP vials located in off-axis and on-axis pressure peaks during sonication. The uncertainty in (c) and (d) represent the standard deviation associated with five independent peak negative pressure and broadband energy measurements, respectively.*

S2 Graphene production methodology

Before comprehensively studying the mechanisms driving the liquid phase exfoliation of graphene, a methodology was developed to ensure the graphene dispersions would be representative of the acoustic cavitation fields that exfoliated them. Aspects of this methodology can be broadly adopted to increase the efficiency of many applications of ultrasonication. For example, users of ultrasonic baths, may find it useful to explore the effect of vial type and geometry on their experiments, and for longer sonication experiments temperature control should be utilised to ensure more consistent sonication; elevated temperatures may actually increase the efficiency of ultrasonication for certain applications.

S2.1 Choice of vial

Low Density Polyethylene, LDPE, Nalgene vials (both purchased from Fisher Scientific) to explore which vial produced the highest graphene yields. It was found that the LDPE vials had a more consistent peak negative pressure HF broadband energy as a function of sonication time (Figure S2.1a) and produced more than double graphene yields with the same processing parameters (3.5 % vs 1.5%). As the vials have different volumes, geometries (not specified by the manufacturer), wall thicknesses, and insertion losses the, vials are not strictly comparable. However, as LDPE has an acoustic impedance that is more closely matched to water than polypropylene vials (PP), this is likely to have a positive effect on the graphene yield due to a decreased perturbation of the acoustic field by the LDPE; water, LDPE and PP have an acoustic impedances of 1.48, 1.79 and 2.4 MRayls, respectively[3].

By chemically wetting vials with Cole Palmer Micro90 surfactant prior to sonication, it was found that the peak negative pressure and the HF broadband energy was more consistent as a function of the sonication time (Figure S2.2a). This is due to the surfactant promoting wetting upon submersion. As such, there will be less trapped gas in the surface micro-cracks of the vials which can dynamically inhibit the transmission of

the acoustic field from the bulk volume of the vessel into the vial. This trapped gas will also nucleate acoustic cavitation outside the vials, which will contribute to the cavitation signals detected by the hydrophone within the vial. As the LDPE vials attenuate the acoustic field, the cavitating bubbles outside the LDPE vial can undergo more intense inertial cavitation and emit a greater amplitude of broadband noise (Figure S2.2b).

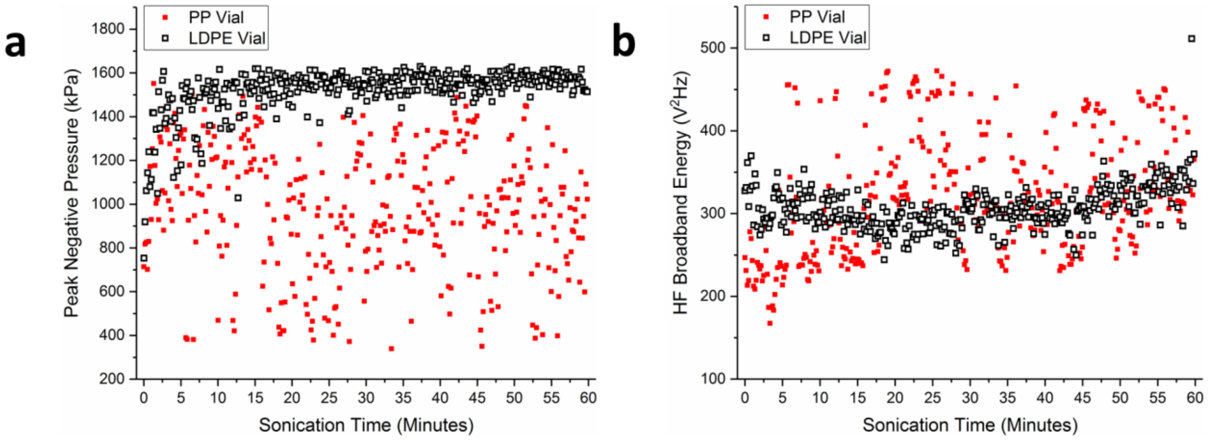

Figure S2.1: *(a) the high-frequency broadband energy and (b) the peak negative pressure with either 15 ml polypropylene vials or 28 ml low density polyethene vials as a function of sonication time. The vials were filled with 3 mg/ml of sodium cholate and 1 mg/ml of graphite and then sonicated at 180 mV_{RMS} with an in-situ HCT hydrophone for 60 minutes. The critical micelle concentration of NaC is ~3.5 mg/ml. Prior to sonication, the vials were chemically wetted with a MICRO-90 surfactant.*

S2.2 Wetting of the vials

The increased inertial bubble activity on the outer walls of the vial can also heat the volume of the vial as well as attenuate the acoustic field entering the vial. Consequently, wetting of vials results in more consistent and reproducible acoustic fields. To ensure wetting of the internal and external walls of the LDPE vials, all samples were prepared at least 12 hours prior to sonication and were soaked in a water-surfactant solution (0.2% by-volume of Cole Palmer MICRO-90).

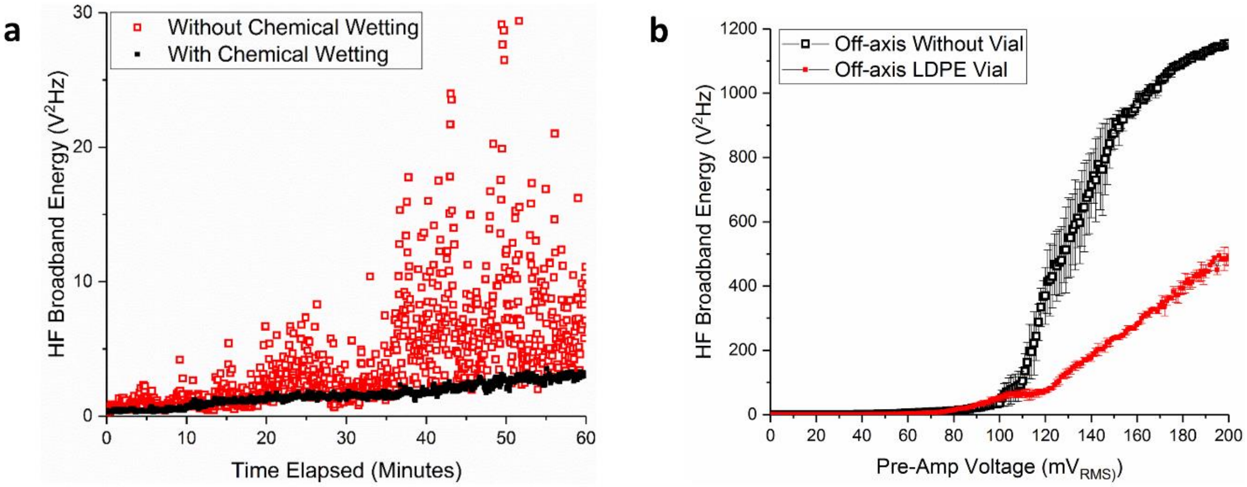

Figure S2.2: *(a) The HF broadband energy as a function of the sonication time in LDPE vials with and without chemical wetting. (b) The HF broadband energy as a function of the pre-amplification voltage. The uncertainty in the broadband energy is associated with the standard deviation of three broadband energy measurements.*

S2.3 Choice of Graphite and Surfactant

To ensure the population of dispersed graphene flakes are representative of the acoustic cavitation mechanisms which exfoliated them, the population of unexfoliated flakes was reduced by optimising the initial graphite concentration. Early experiments with high initial concentrations (50 mg ml^{-1}) of graphite had graphene yields of only 0.1%. By varying the initial concentration of graphite, it was found that the yield could be increased by lowering the initial graphite concentration to 0.2 mg ml^{-1}. As cavitation only interacts with the top surface of graphite flakes [4], having isolated graphite flakes during sonication will increase the population of graphite flakes being exfoliated simultaneously, whereas with a dense layer of graphite flakes only the top layer of flakes will be exfoliated. As such, lower initial concentrations of graphite will increase the population of graphite being exfoliated simultaneously and therefore increase the resulting dispersed graphene yields. However, for large scale production this methodology is not practical, as a low initial graphite concentration will limit the overall exfoliation rate[5]. To further optimise the graphene yield, an array of graphite grades and surfactants were explored.

Graphite grades with distinct size populations were purchased from Asbury Carbons:

Microfine:	98%	< 325 Mesh	(44 micron)
Fine Flake:	80%	< 100 Mesh	(150 micron)
#1 Flake:	>85%	> 80 Mesh	(180 micron)

These graphite grades were sonicated with a water-surfactant solution containing either sodium cholate (NaC) or sodium deoxycholate (NaDOC), which are two commonly used surfactants in the liquid phase exfoliation literature[5-7]. The graphite samples were sonicated in LDPE vials over 60 minutes at a pre-amp voltage of 140 mV_{RMS}. The initial graphite and surfactant concentration were 0.2 and 2 mg ml^{-1}, respectively. Figure S2.3a shows that the graphene yield significantly increased when sonicating graphite grades containing larger graphite flakes. Of the two surfactants, it was also found that sodium cholate produced higher graphene yields across all the graphite grades.

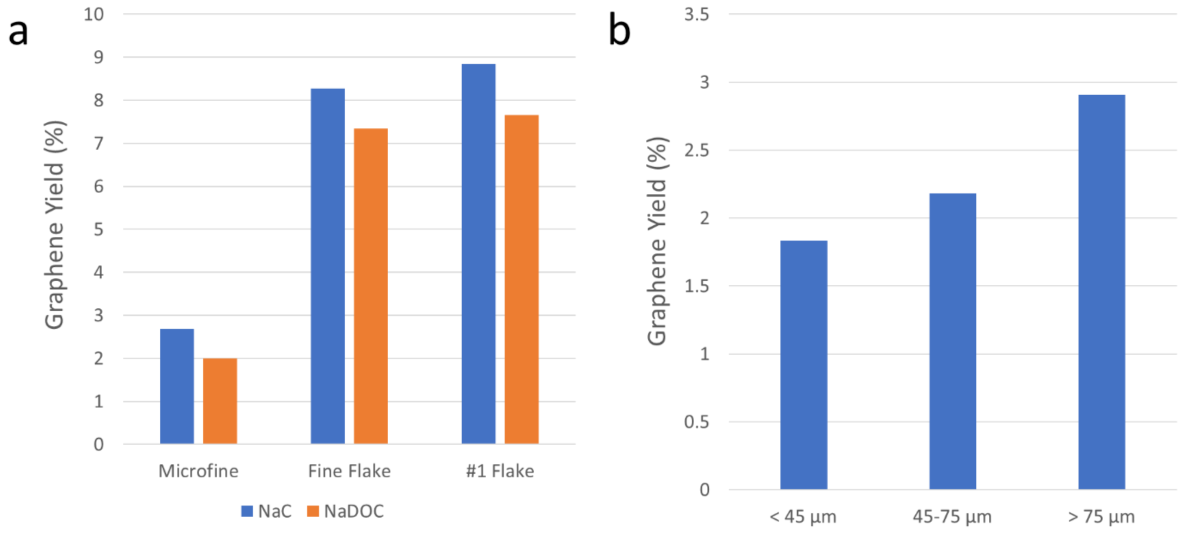

Figure S2.3: *(a) The graphite yield as a function of the graphite grade and surfactant type and (b) The graphene yield of Asbury Carbons Fine Flake graphite that was sieved to different size distributions using a pair of 45 μm and 75 μm test sieves.*

To ensure the size distributions of the graphene dispersions were representative of the acoustic cavitation mechanisms exfoliating them, the initial graphite population was sieved to a size range of 45-75 μm using a 45 μm and a 75 μm test sieve; the Asbury Carbons Fine Flake graphite grade was chosen to optimise the yield of sieved graphite through the test sieves. Although the use of flakes larger than 75 μm resulted in a higher graphene yield (Figure S2.3b), having a known and relatively narrow size distribution of 45-75 μm ensured that size distribution trends in the graphene dispersions should be more consistent and reflective of the acoustic cavitation mechanisms that exfoliated them. However, due to the inefficiency of sieving (13% yield of 45-75 μm graphite sieved from the Fine Flake graphite grade), this processing technique may not scalable as it is relatively time consuming and wasteful. Figure S2.4 demonstrates that the experimental methodology has resulted in very efficient and relatively homogenous exfoliation such that the entire initial graphite population has been in to sub-micron flakes.

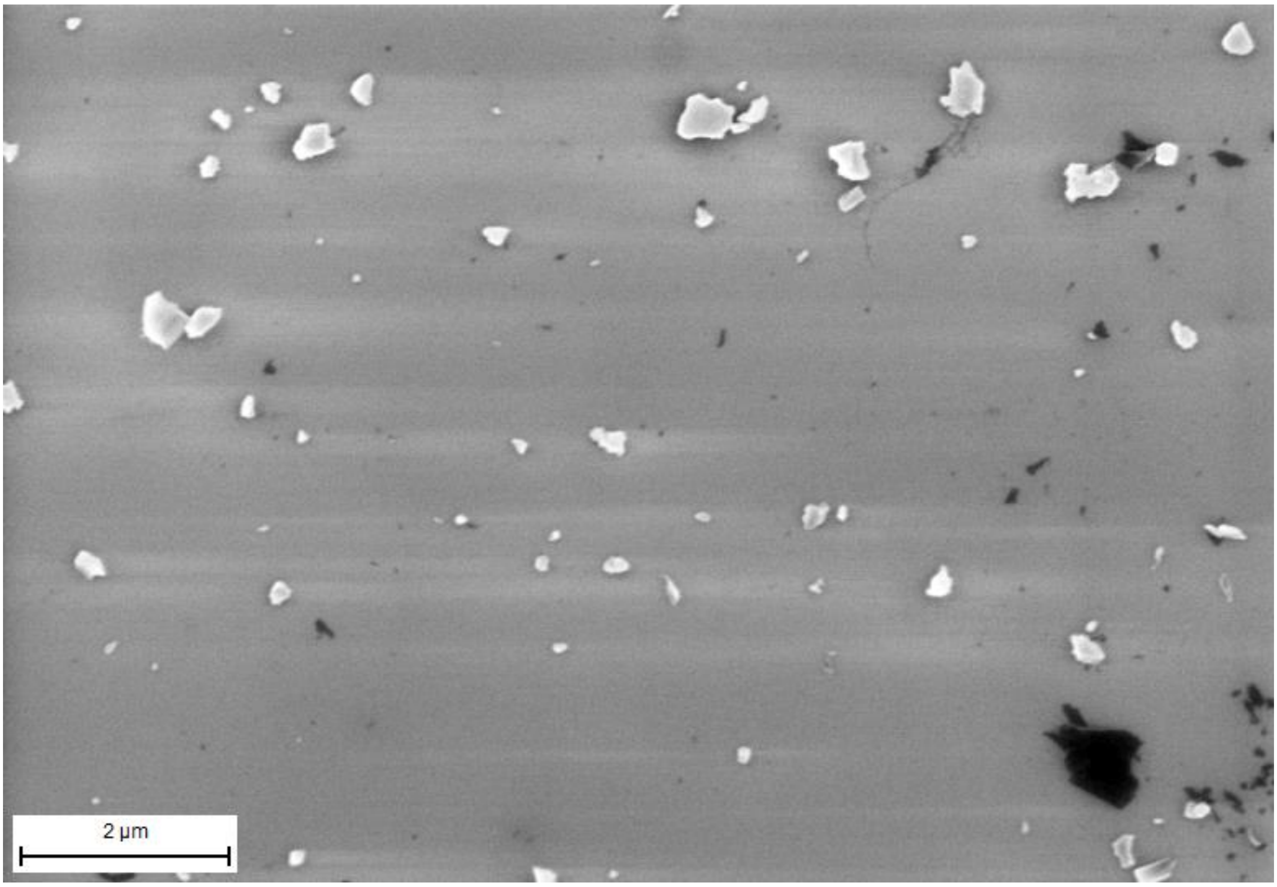

Figure S2.4: *A representative SEM micrograph of the precipitate that remained after removing the supernatant.*

S2.4 Cooling strategy

During graphene production, it was found that the graphene dispersions produced over long sonication times produced anomalous results. Figure S2.5a shows that the graphene produced over long 3 and 4-hour sonication times had graphene yield trends that were distinctly different to those produced over shorter (<120 minutes) sonication times. It was hypothesised that the temperature build-up during sonication (Figure 2.5b) could be affecting the graphene exfoliation rate. This was suggested by Kim *et al.*[8] and confirmed experimentally (Figure S2.6) by producing graphene samples with and without actively cooling the vessel with an array of ten 12 V pc fans.

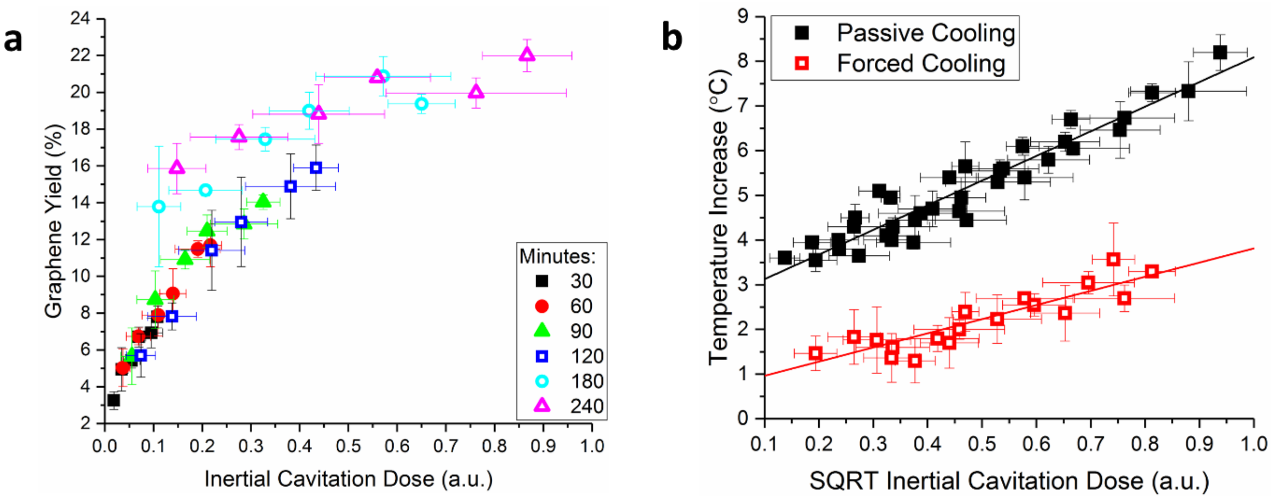

Figure S2.5: *(a) The graphene yield as a function of the ICD when the vessel was not actively cooled. (b) The temperature increase inside the LDPE vials during sonication as a function of the ICD. The uncertainty in the graphene yield and the ICD is associated with the standard deviation of three graphene yield measurements and five broadband energy measurements, respectively.*

Over relatively short 60-minute sonication times, it was found that the graphene yield was independent of the cooling strategy (Figure S2.6a), whereas over longer 180-minute sonication times the graphene yield was dependent on the cooling strategy used (Figure S2.6b). Accordingly, the anomalous samples produced over 120-minute sonication times were re-produced using shorter 30-minute sonication time intervals

while also actively cooling the reference vessel. Therefore, using active cooling, decreasing the sonication time or using pulsed mode (rather than continuous wave) ultrasound will help mitigate the temperature increase during sonication and will ensure more consistent results. Furthermore, depending on the application sonication at elevated temperatures may increase the efficiency of ultrasonication (Figure S2.5a), however temperature control will still be required to achieve this consistently.

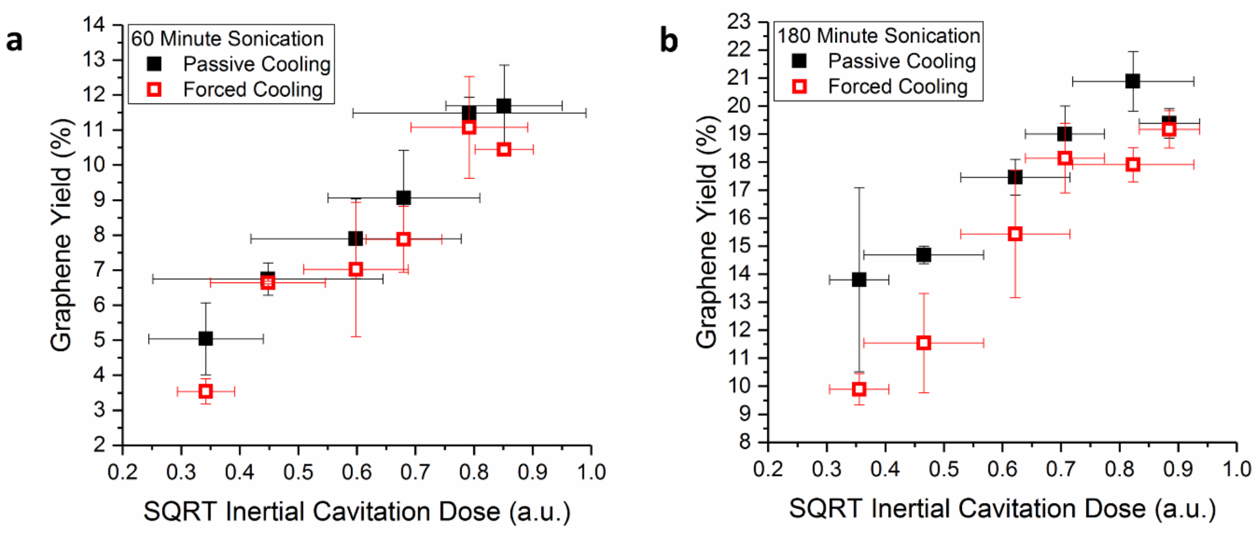

Figure S2.6: *The graphene yield as a function of the square root of the square root of the ICD with (a) and without (b) actively cooling with an array of 12 V pc fans. The uncertainty in the graphene yield and the ICD is associated with the standard deviation of three graphene yield measurements and five broadband energy measurements, respectively.*

S3 Alternative acoustic cavitation metrics

Although there are many accepted measurement standards for quantifying the primary effects of ultrasound (frequency, intensity, power, *etc.* [9]), there is a wide range of non-standardised techniques for detecting and quantifying acoustic cavitation [10]. These measurement techniques include, measuring the acoustic emissions from cavitating bubbles using hydrophones[11] and cavitation sensors [12, 13], measuring the light emission from cavitating bubbles (sonoluminescence) [14, 15], measuring the temperature increase

during sonication (calorimetry) [16, 17], and measuring the cavitation driven erosion of surfaces such as aluminium foil [18, 19]. However, due to the absence of established measurement standards and broad unfamiliarity with acoustic cavitation, purely extrinsic parameters such as temperature calorimetry [17] and nominal electrical input power [20] are commonly used to monitor and develop ultrasonication methodologies.

Temperature calorimetry measures the heat build-up that is generated by the quasi-adiabatic collapses of acoustic cavitation (Figure S1.6b). As such, the graphene yield was found to rise as a function of the temperature increase (Figure S3.1a). However, due to the non-uniform nature of cavitation fields and the high specific heat capacity of water, temperature calorimetry cannot quantify the real-time activity of acoustic cavitation and has no spatial sensitivity. Furthermore, temperature calorimetry can be unreliable as changes in the ambient conditions and or the operating parameters, such as nominal electrical/acoustic power or sonication time, can skew temperature calorimetry measurements due to the transducers heating up during operation. This is shown in Figure S2.6 and Figure S3.1a, where the graphene yield is dependent on the cooling strategy used.

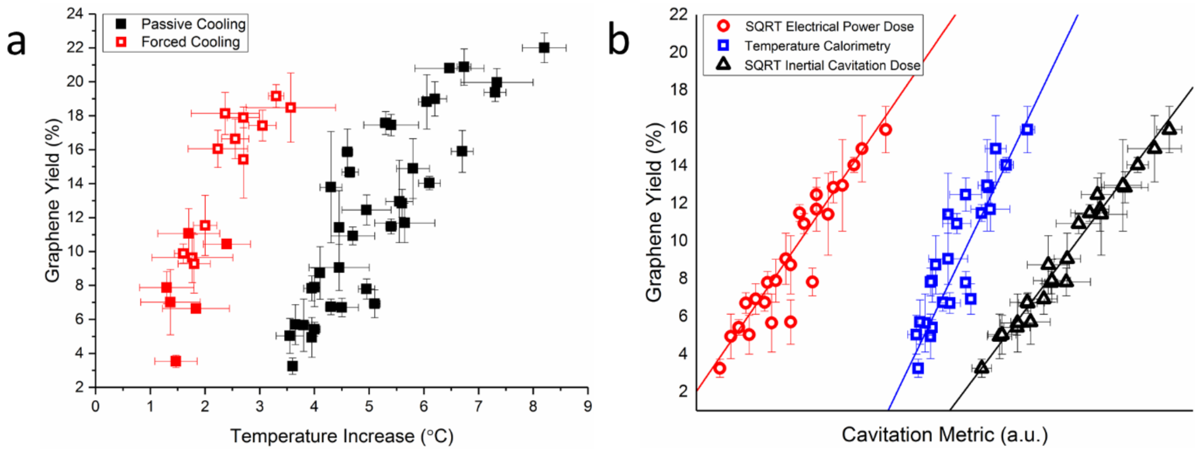

Figure S3.1: *(a) The graphene yield as a function of the temperature increase within LDPE vials and cooling strategy. (b) The graphene yield as a function of three distinct normalised acoustic cavitation metrics. Uncertainties represent the standard deviation of three measurements, except for the ICD which is associated with five broadband energy measurements.*

Figure S3.1b shows the graphene yield as a function of three distinct cavitation metrics, the electrical power dose, temperature calorimetry and the inertial cavitation dose (ICD). Where the electrical power dose (analogous to ICD) is the electrical power delivered to the reference vessels transducer ring (read from the E&I power amplifier) multiplied by the sonication time. Of the three acoustic cavitation metrics, Figure S3.1b demonstrates that the ICD is the most reliable metric for predicting graphene yield as it had the highest Pearson's R (0.98) and R-Square (0.97) values (Table S3.1). This is because the ICD is a direct measurement of the real-time acoustic signals from cavitating bubbles whereas temperature calorimetry and nominal input power are indirect measurements of acoustic cavitation. When utilising NPL's cavitation sensors[21], the inertial cavitation field is spatially resolvable, whereas the temperature calorimetry offers a limited spatial resolution (Figure S1.2) and electrical power dose includes no spatial resolution data. Furthermore, as inertial cavitation activity is highly non-linear with nominal input power (Figure 1d), electrical power dose is increasingly unreliable as an inertial cavitation metric over broad nominal input power ranges. However, with a fixed power output, electrical power dose has been demonstrated to correlate with the graphene exfoliation rate[20].

Table 3.1: Linear fit parameters of the acoustic cavitation metrics shown in Figure S3.1b.

Plot	Electrical Power Dose	Temperature Calorimetry	Inertial Cavitation Dose
Equation	y = a + b*x	y = a + b*x	y = a + b*x
Intercept	-25.4 ± 3	-1.6 ± 0.7	-20.1 ± 0.9
Slope	26.1 ± 2.3	18.2 ± 1.1	16.6 ± 0.5
Residual Sum of Squares	88.97	51.90	13.95
Pearson's r	0.92	0.96	0.99
R-Square(COD)	0.85	0.91	0.98
Adj. R-Square	0.85	0.91	0.98

Alongside exploring power and temperature calorimetry acoustic cavitation metrics, the effect of the MHz frequency band on the correlation between the square root of the ICD and the graphene yield (Figure 2b)

was explored. This was carried out by calculating broadband energy over a wide range of 0.5 MHz frequency bands as a function of pre-amp voltage. Figure S3.2a shows that most of the high frequency noise generated by inertial cavitating bubbles is contained within a frequency band of 1.5 -2.5 MHz. This sensitivity is likely due to a combination of the frequency sensitivity of the hydrophone and the drive electronics, as well as the acoustic impedance of the LDPE vials.

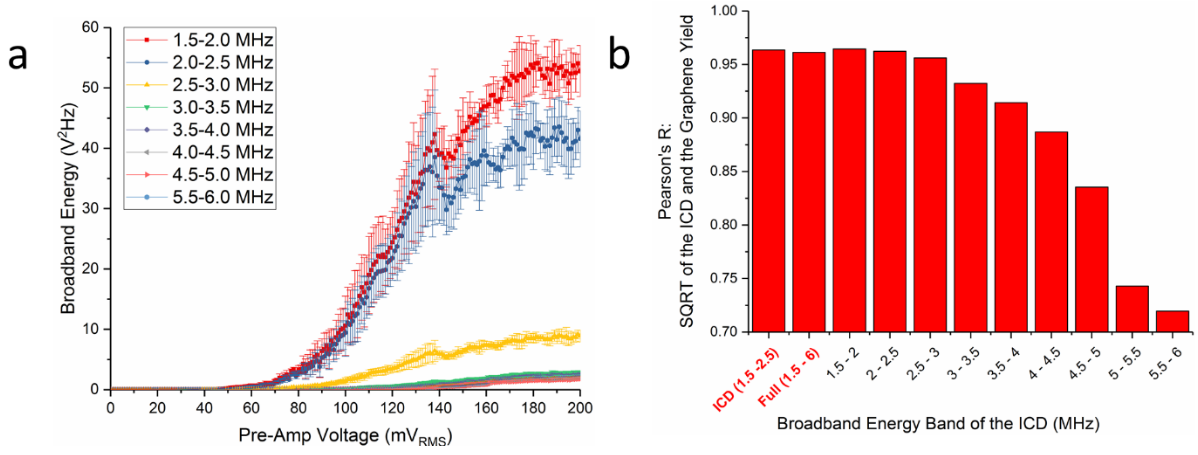

Figure S3.2: *(a) The broadband energy as a function of the frequency band over which the ICD is calculated and the pre-amp voltage. (b) The Pearson's R of the Square root of the ICD and the graphene yield as a function of the broadband energy band. Pearson's R values are also shown for the ICD (1.5 – 2.5 MHz) and the full bandwidth of the high frequency spectra that were collected during cavitation measurements (1.5 – 6 MHz).*

As the high frequency noise is mostly isolated within a 1.5 - 2.5 MHz band (Figure S1.1b), this frequency band was initially used to calculate the ICD. Figure S3.2b shows that the correlation (calculated using Pearson's R) between the graphene yield and the square root of the inertial cavitation dose decreases when calculating the dose over higher frequency bands. As such, the graphene yield is more strongly correlated with the intense broadband noise that is generated over 1.5 – 2.5 MHz. As the 1.5 – 2.5 MHz noise dominates the high frequency spectra (Figure S3.2a), all ICD's which contained this significant broadband noise had the highest Pearson's R values. Interestingly, the slight peak in the high frequency spectra that is observed around 5.5 MHz (Figure S1.1b) which could be attributed to inertial cavitation

activity does not increase the Persons R's values for ICD's calculated around 5 MHz. This suggests the 5 MHz peak is not directly related to inertial cavitation activity and may arise from the frequency sensitivity of the drive electronics/hydrophone. Figure S3.2 further demonstrates that inertial cavitation, drives graphene exfoliation during sonication, and by optimising the ICD to the frequency sensitivity of the experimental setup, the ICD can be used to better predict the inertial cavitation driven liquid phase exfoliation of graphene.

S4 Characterisation Methods

S4.1 UV-Vis Spectroscopy

UV-Vis Spectroscopy was carried out using a Perkin Elmer Lambda 850 spectrophotometer. Graphene samples were diluted to an optical density of ~0.5 (~0.01 mg ml^{-1}) using de-ionised water prior to UV-Vis analysis. The UV-Vis absorbance of the diluted graphene samples was analysed over a spectral wavelength range of 250-800 nm, in a self-masking Helma quartz cuvette with a 10 mm path length. Due to the scattering contributions of graphene, the absorbance measured by the spectrophotometer is a measurement of extinction rather than absorbance[22]. Representative UV-Vis extinction spectra are shown in Figure S4.1a. The dispersed concentration of graphene was calculated using the Beer-Lambert-law with a size-independent extinction coefficient[23] of $\varepsilon_{750} = 5450\ L\ g^{-1}m^{-1}$. Figure S4.1b shows the ratio of the UV-Vis peak to the long wavelength plateau, $\varepsilon_{550}/\varepsilon_{max}$, as function a of the square root of the inertial cavitation. As this ratio is proportional to the mean graphene length [23], the linear correlation in Figure S4.1b is consistent with the quantitative SEM length distributions (Figure 2e) discussed in the main text.

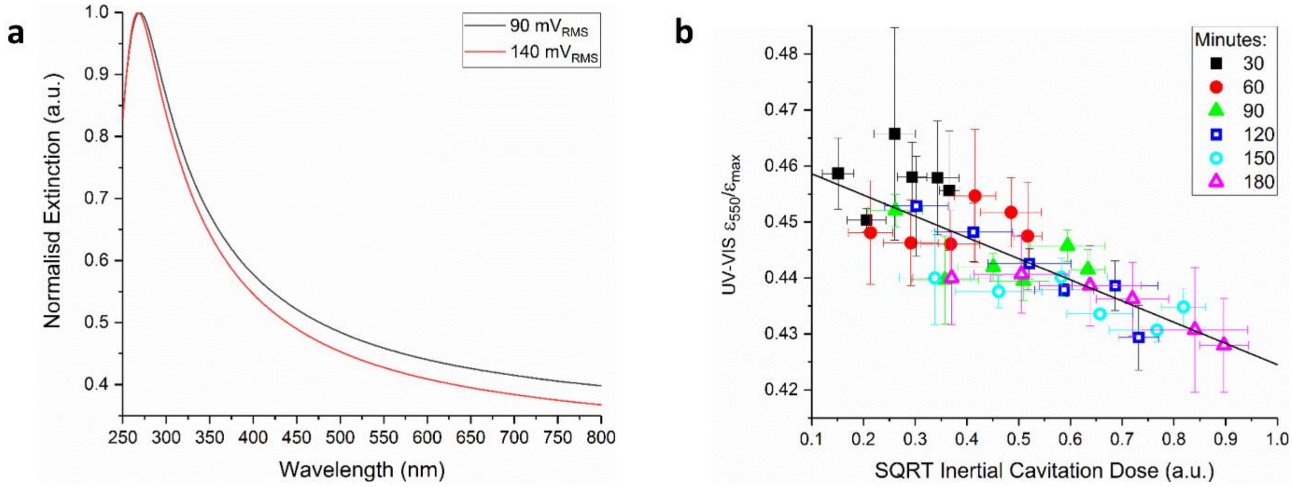

Figure S4.1: *(a) Normalised extinction spectra of graphene dispersions produced at the minimum and maximum of the ICD range. (b) The* $\varepsilon_{550}/\varepsilon_{max}$ *extinction ratio as a function of the square root of the ICD. The uncertainty in the* $\varepsilon_{550}/\varepsilon_{max}$ *measurement is associated with the standard deviation of three measurements.*

S4.2 Raman Spectroscopy

Raman spectrometry was carried out using a Renishaw inVia confocal Raman microscope with 532 nm excitation in air. The Raman microscope is located in a 20℃ temperature-controlled room. The Raman spectra were collected using in a 100x objective lens (spot size ~ 1 μm) using 10% of the laser power (~0.46 mW). Graphene samples were prepared by diluting graphene dispersions to an optical density of ~0.5 (~0.01 mg ml^{-1}). 5 ml of the diluted dispersions was filtered through 25 mm diameter Whatman Anodisc inorganic alumina membranes (20 nm pore size), purchased from Sigma Aldrich, using vacuum filtration. 5 ml of IPA was then filtered through the membranes to remove residual surfactant. Mapping of the alumina membranes was carried out over a 20 x 20 μm^2 area in 2 μm steps. By having a one second acquisition time and 10% laser power, the re-stacked graphene films were not visibly damaged by the laser excitation. The 120 spectra were baseline corrected, averaged, and normalised to the G peak intensity before being analysed.

To measure the variations on each re-stacked graphene film, spectral measurements were also performed on the individual spectra captured across each 2D Raman map. Within each 2D map, there were large

fluctuations in spectral measurements such as I_D/I_G (Figure S4.2a) and I_{2D}/I_G (Figure S4.2b) suggesting that there was a large variance in the graphene population in each graphene dispersion. This is likely due to the relatively low centrifugation rates (1000 rpm, 120g) that were chosen to minimise the removal of larger graphene flakes during centrifugation. Accordingly, these findings suggest that Raman size distribution metrics can be unreliable in graphene dispersions with relatively wide size distributions.

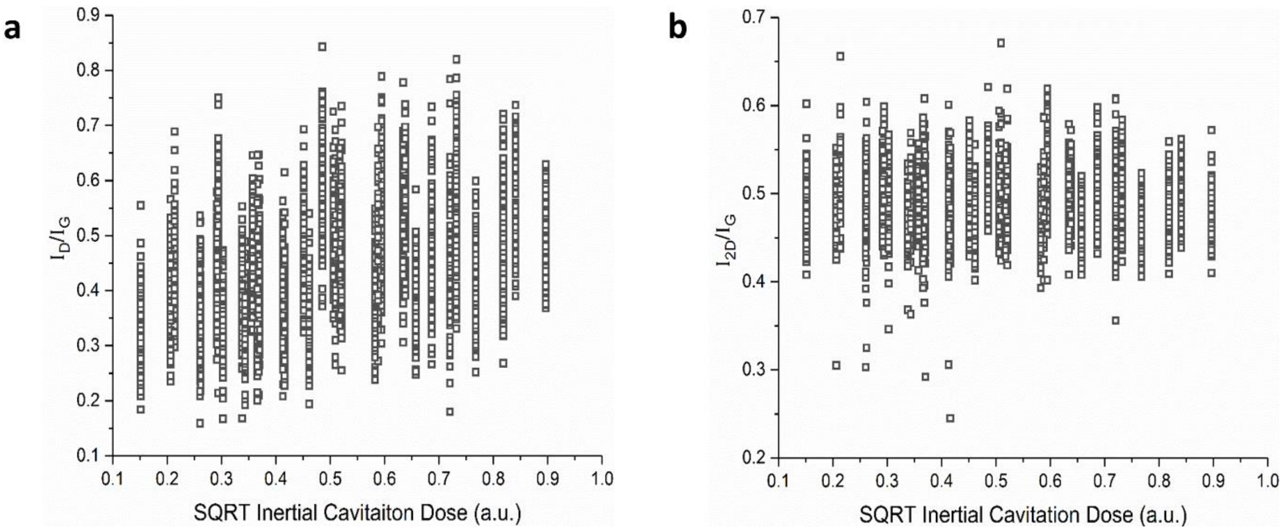

Figure S4.2: *The I_D/I_G (a) and I_{2D}/I_G (b) ratios from the 120 individual spectra that were captured across each re-stacked graphene film, as a function of the square root of the ICD.*

Beyond I_D/I_G and I_{2D}/I_G ratios, Backes *et al.*[23] demonstrated that other spectroscopic Raman metrics are indicative of nanosheet size. These include calculating the ratio of the graphite 2D peak maximum intensity (2720 cm^{-1}, Figure S4.3) to its 2D peak shoulder (~2690 cm^{-1}, Figure S4.3), and the full width at a half maximum (FWHM) of the G peak [23]. Both these metrics (Figure S4.4 a,b) were found to be consistent with the quantitative SEM (Figure 2a) and AFM (Figure 2b) size distribution analysis discussed in the main text.

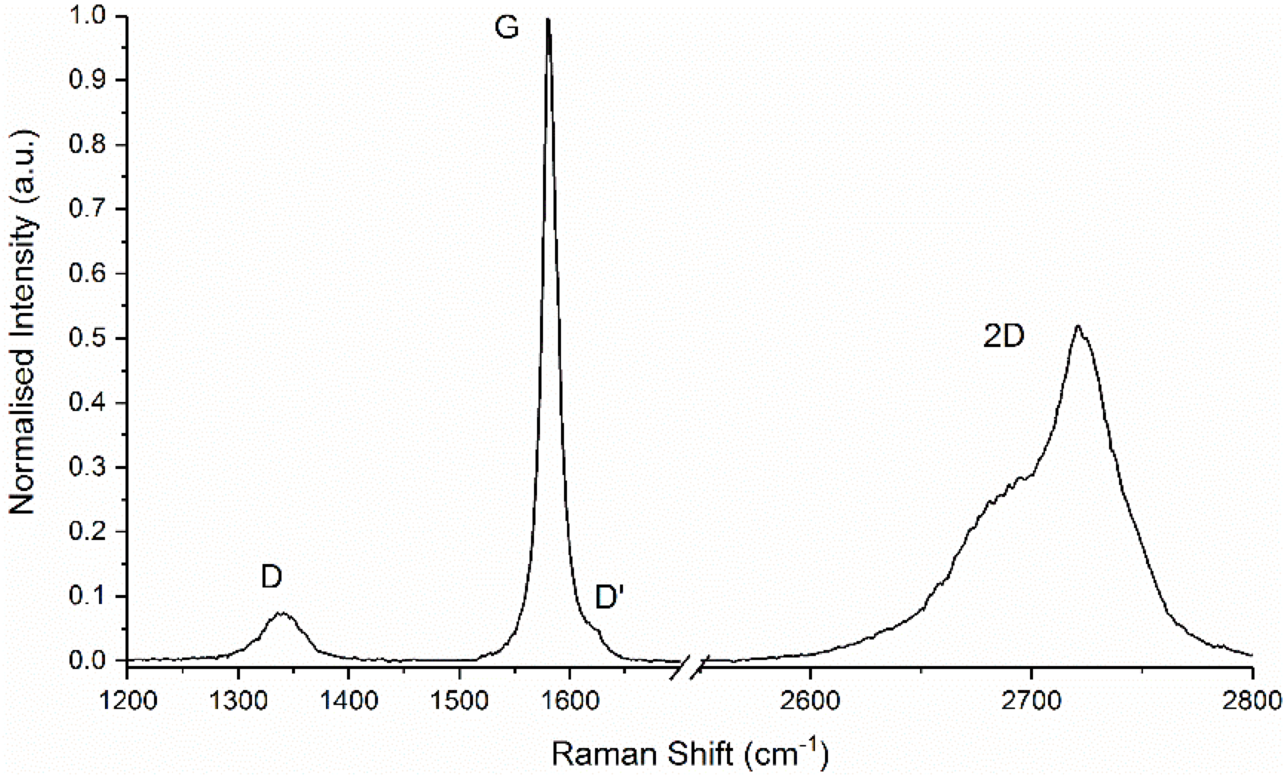

Figure S4.3: *The Raman spectrum of the pre-treated and sieved graphite used in this work.*

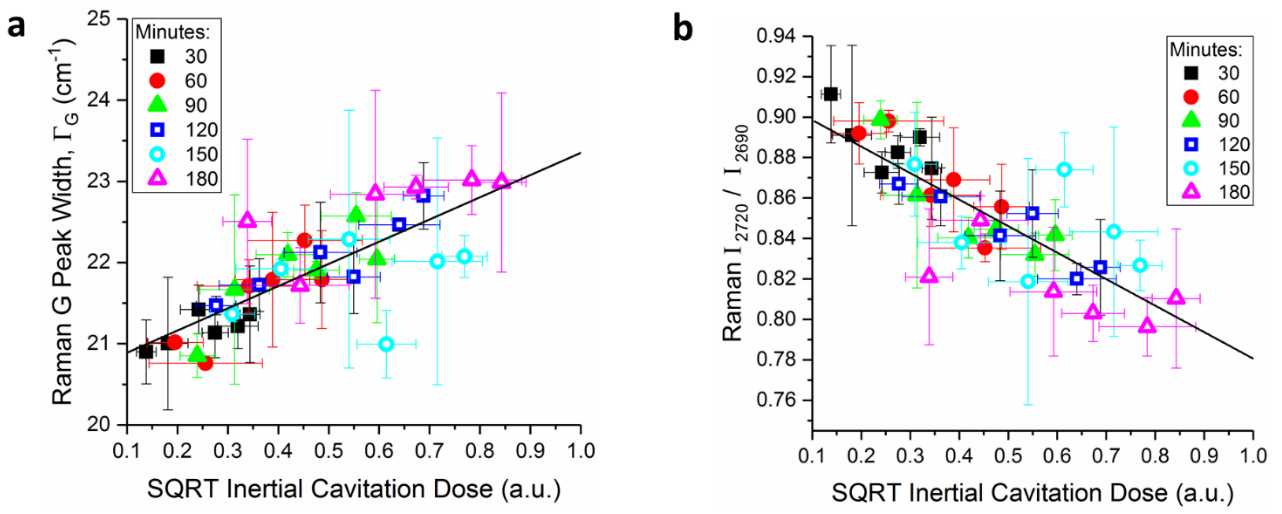

Figure S4.4: *(a) The full width at half maximum of the Raman G Peak as a function of the square root of the ICD. (b) The ratio of the graphite 2D peak (~2720 cm^{-1}) to the graphite 2D peak shoulder (~2690 cm^{-1}) as a function of the square root of the ICD. The uncertainty in the I_D/I_G ratio, Γ_G, I_{2720}/I_{2960}, and the ICD is associated with the standard deviation of three Raman measurements and five broadband energy measurements per sample, respectively.*

S4.3 Atomic force microscopy

Atomic Force Microscopy, AFM, was carried out using an Asylum Research MFP 3D using Nanosensors PPP-NCHR tips, purchased from Windsor Scientific. Graphene dispersions were analysed on silicon wafers with a 300 nm wet thermal oxide, purchased from University Wafer. The wafers were cut into 10 x 10 mm substrates and were cleaned by sonicating them for ten minutes in acetone, IPA and then DI water. The substrates were then further cleaned in a UV-Ozone cleaner for 10 minutes. Graphene dispersions were then diluted to an optical density of 0.25 (at 750 nm, ~0.005 mg ml^{-1}). 40 μl of these diluted graphene dispersions was drop casted, in 10 μl increments, on to cleaned Si/SiO_2 substrates which were heated to 150°C using a hotplate. To remove excess surfactant, the substrates were then rinsed with approximately 2 ml of DI water and 2 ml of IPA, before being dried with compressed air. This sample preparation method ensured homogeneous coverage of graphene flakes while maintaining suitable flake separation (Figure S4.5).

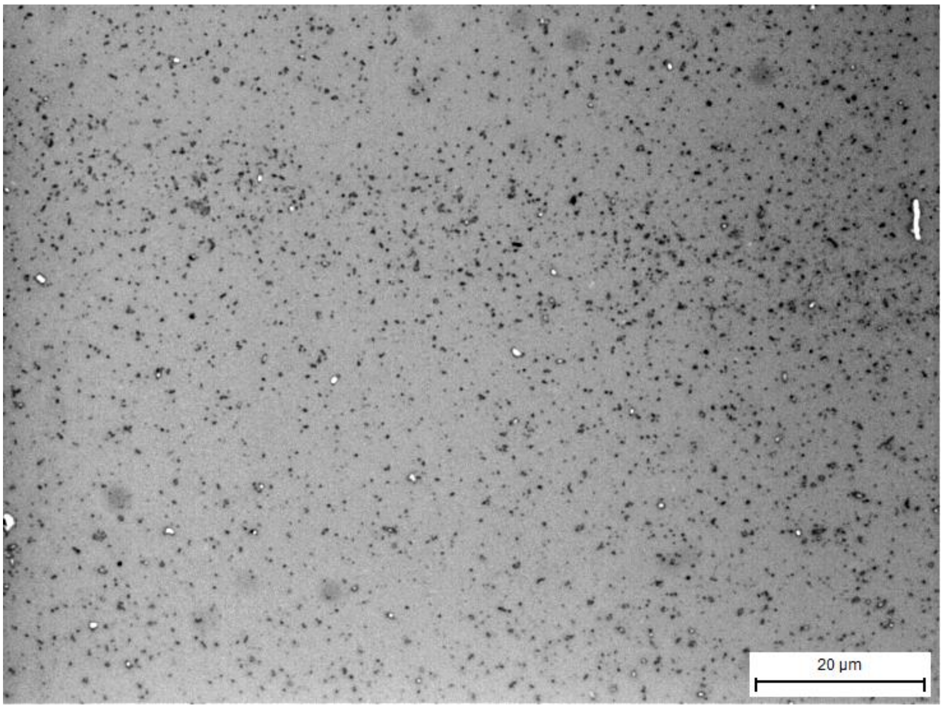

Figure S4.5: *An optical microscopy image of graphene deposited on Si/SiO_2 wafers, taken with an Olympus CX40 optical microscope with a 50X objective.*

AFM microscopy was carried out by scanning large 20 x 20 μm areas to find 2 x 2 μm regions with dense flake coverage while maintaining flake separation. These areas were then scanned with a 512 x 512 resolution using a low scan speed (2 μm s^{-1}) and a relatively high set point (280) to minimise damage to the tip and the sample. The resultant AFM images (Figure S4.6a) were then analysed using SPIP. To minimise distortion to the measured height distributions, the only image modification carried out by SPIP was to manually level the background. The apparent heights of the flakes were measuring using SPIP's height profile tool. The profile tool was used in the AFM scanning direction to ensure accurate height measurements (Figure S4.6a). Flake heights were made by using a cursor tool, shown in Figure S4.6b, to subtract the flake height from the mean height of the background. Three of these flake height measurements were carried out across each flake to get a mean height of each flake. To get a representative thickness distribution at least 100 flakes were analysed from each graphene sample analysed using this methodology.

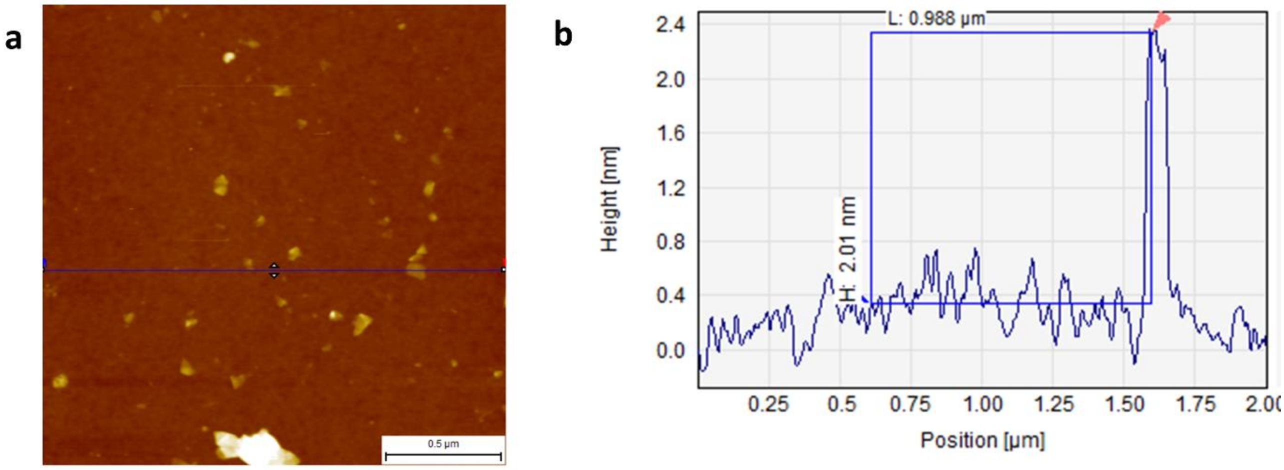

Figure S4.6: *(a) A representative AFM image of graphene deposited on an Si/SiO$_2$ substrate. The blue line is an X-axis height profile tool, the data of which is shown in (b).*

As the height measured by AFM does not account for the height contribution of adsorbed surfactant, it does not reflect the true thickness. To convert the measured flake height (in nanometers) to thicknesses (in layers), a relationship reported by Paton *et al.*[24] was used. By carrying out a detailed step-height analysis of

graphene terraces, Paton *et al.*[24] found that one layer of surfactant-exfoliated graphene contributes 0.95 nm to the overall height of a multi-layered graphene flake. Furthermore, monolayer graphene has an apparent AFM height of 2 nm due to a ~nanometer of adsorbed surfactant; a finding which was confirmed by Raman spectroscopy. As such the number of layers, L, in graphene flakes was calculated from their apparent AFM heights, h, by using the following relation:

$$L = \left(\frac{h - 1.05}{0.95}\right)$$

Due to Paton et al.[24] using the same surfactant and silicon wafer type[24], this relationship was directly applicable to this study.

S4.4 Scanning Electron Microscopy

Scanning Electron Microscopy, SEM was carried out on a Zeiss Supra 40. Graphene samples for SEM analysis were prepared in the same way as for AFM analysis. SEM analysis was carried out using an accelerating voltage of 5 kV using the microscopes built-in InLens detector. SEM micrographs (Figure S4.6) were captured at a magnification of 40 kX with an image resolution of 2048 x 1536 pixels. To minimise surface charging, SEM micrographs were captured with a high scan speed. To decrease noise, 100 scans were accumulated for each micrograph, and a pixel average was performed. SEM micrographs were analysed using SPIP following NPLs good practice guide on graphene characterisation[25]. The graphene size distributions were measured by using SPIP's calliper tools; pictured in Figure S4.7. To get a representative size distribution 300 – 400 flakes were analysed from each graphene sample, and the arithmetic mean, and uncertainty (standard error of the mean) was calculated. Alongside the length measurement in the main text, a width measurement was also made simultaneously (Figure S4.7). Like the length measurement, the mean graphene width decreases as a function of the square root of the inertial cavitation (Figure S4.8).

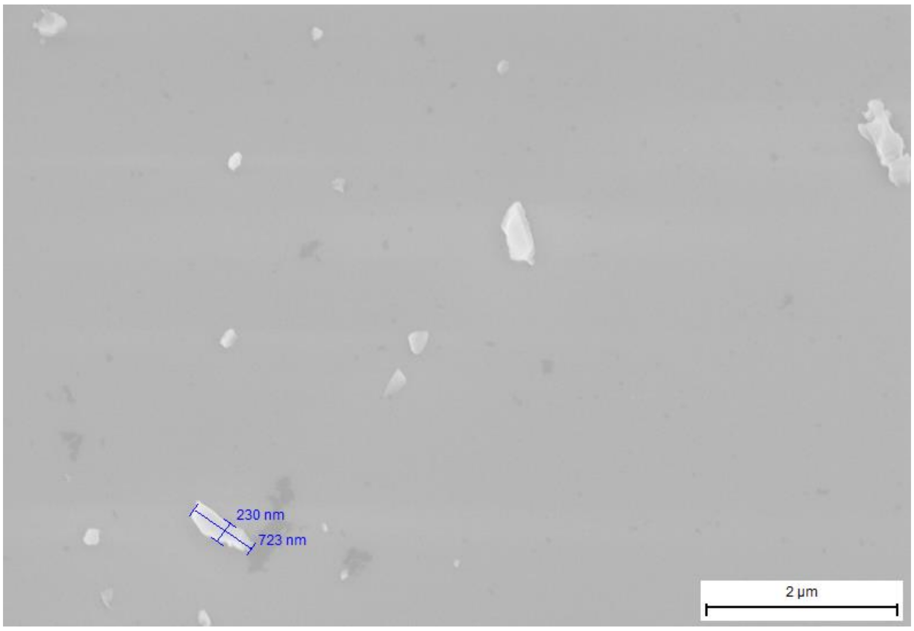

Figure S4.7: *Representative SEM micrograph of graphene deposited on a Si/SiO_2 substrate. Pictured are the SPIP calliper tools that were used to measure the lengths and widths of graphene flakes.*

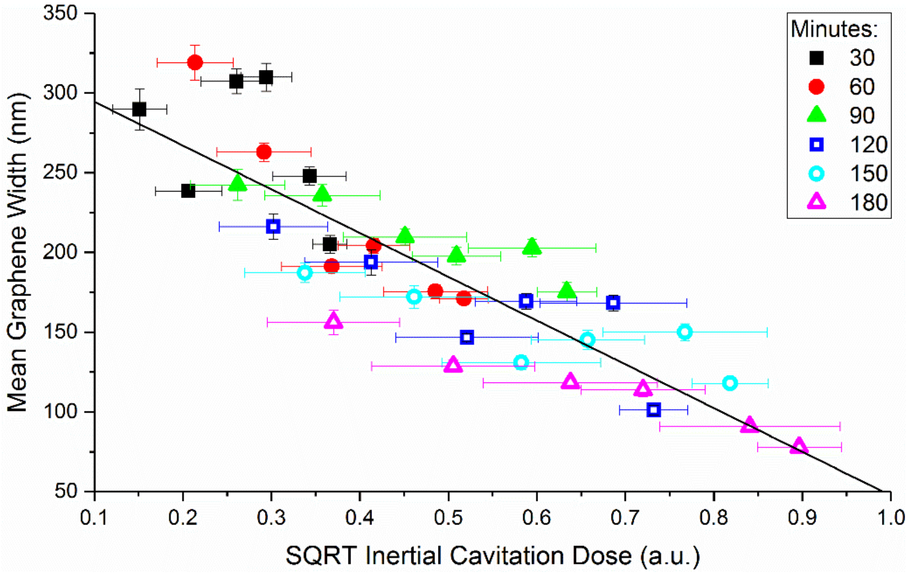

Figure S4.8: *The mean graphene width as a function of the square root of the ICD. The uncertainty in the ICD is associated with the standard deviation of five broadband energy measurements, and the uncertainty in the graphene width is associated with the standard error of ~300 measurements.*

S5 Additional Graphs

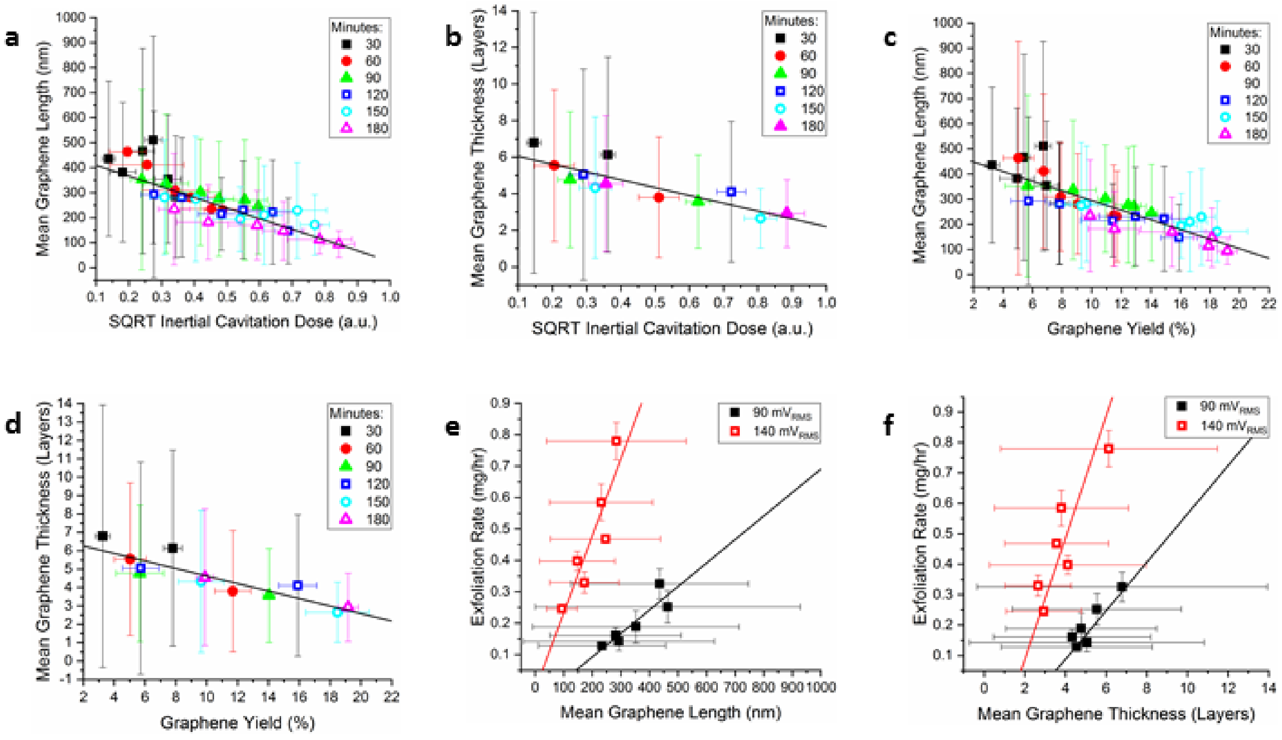

Figure S5.1: *The mean graphene (a) length and (b) thickness as a function of the ICD. The mean graphene (c) length and (d) thickness as a function of the graphene yield. The symbols in (a-d) delineate the data as a function of sonication time, measured in minutes. The graphene exfoliation rate ($c_g\ t^{-1}$) as function of (e) the mean graphene length and (f) thickness for graphene samples produced with the highest and lowest pre-amp voltages (acoustic powers) used in this work. The uncertainty in the graphene yield and exfoliation rate is associated with the standard deviation of three graphene yield measurements at each sonication time, and the uncertainty in graphene length and thickness is associated with the standard deviation of ~300 and ~100 measurements, respectively.*

References

1. Hodnett, M. & Zeqiri, B. Toward a reference ultrasonic cavitation vessel: Part 2-Investigating the spatial variation and acoustic pressure threshold of inertial cavitation in a 25 kHz ultrasound field. *Ultrasonics, Ferroelectrics, and Frequency Control, IEEE Transactions on* **55**, 1809-1822 (2008).

2. Wang, L. *et al*. Towards a reference cavitating vessel Part III—design and acoustic pressure characterization of a multi-frequency sonoreactor. *Metrologia* **52**, 575 (2015).

3. Electronics Engineers. Sonics, Ultrasonics Group & Institute of Radio Engineers. Professional Group on Ultrasonics Engineering. *IEEE Transactions on Sonics and Ultrasonics*, Institute of Electrical and Electronics Engineers, 1970).

4. Xia, Z. Y. *et al*. The exfoliation of graphene in liquids by electrochemical, chemical, and sonication-assisted techniques: A nanoscale study. *Advanced Functional Materials* **23**, 4684-4693 (2013).

5. Ramalingam, P., Pusuluri, S. T., Periasamy, S., Veerabahu, R. & Kulandaivel, J. Role of deoxy group on the high concentration of graphene in surfactant/water media. *RSC Advances* **3**, 2369-2378 (2013).

6. Smith, R. J., Lotya, M. & Coleman, J. N. The importance of repulsive potential barriers for the dispersion of graphene using surfactants. *New Journal of Physics* **12**, 125008 (2010).

7. Lotya, M., King, P. J., Khan, U., De, S. & Coleman, J. N. High-concentration, surfactant-stabilized graphene dispersions. *ACS Nano* **4**, 3155-3162 (2010).

8. Kim, J. *et al*. Direct exfoliation and dispersion of two-dimensional materials in pure water via temperature control. *Nature communications* **6**, 8294 (2015).

9. International Electrotechnical Commission. Ultrasonics - Hydrophones - Part 1: Measurement and characterization of medical ultrasonic fields up to 40 MHz. *IEC 62127-1* (2007).

10. Gallego-Juárez, J. A. & Graff, K. F. in *Power Ultrasonics: Applications of High-Intensity Ultrasound, Chapter 8* (Elsevier, 2014).

11. Leighton, T. G. A strategy for the development and standardisation of measurement methods for high power/cavitating ultrasonic fields: review of cavitation monitoring techniques. (1997).

12. Zeqiri, B., Gelat, P. N., Hodnett, M. & Lee, N. D. A novel sensor for monitoring acoustic cavitation. Part I: Concept, theory, and prototype development. *Ultrasonics, Ferroelectrics, and Frequency Control, IEEE Transactions on* **50**, 1342-1350 (2003).

13. Hodnett, M., Chow, R. & Zeqiri, B. High-frequency acoustic emissions generated by a 20 kHz sonochemical horn processor detected using a novel broadband acoustic sensor: a preliminary study. *Ultrason. Sonochem.* **11**, 441-454 (2004).

14. Lauterborn, W., Kurz, T., Geisler, R., Schanz, D. & Lindau, O. Acoustic cavitation, bubble dynamics and sonoluminescence. *Ultrason. Sonochem.* **14**, 484-491 (2007).

15. Lee, J. *et al*. Spatial distribution enhancement of sonoluminescence activity by altering sonication and solution conditions. *The Journal of Physical Chemistry B* **112**, 15333-15341 (2008).

16. Son, Y., Lim, M., Khim, J., Kim, L. & Ashokkumar, M. Comparison of calorimetric energy and cavitation energy for the removal of bisphenol-A: the effects of frequency and liquid height. *Chem. Eng. J.* **183**, 39-45 (2012).

17. Buzaglo, M. *et al*. Top-Down, scalable graphene sheets production: It is all about the precipitate. *Chemistry of Materials* **29**, 9998-10006 (2017).

18. Jüschke, M. & Koch, C. Model processes and cavitation indicators for a quantitative description of an ultrasonic cleaning vessel: Part I: Experimental results. *Ultrason. Sonochem.* **19**, 787-795 (2012).

19. Koch, C. & Jüschke, M. Model processes and cavitation indicators for a quantitative description of an ultrasonic cleaning vessel: Part II–Multivariate data analysis. *Ultrason. Sonochem.* **19**, 796-802 (2012).

20. Buzaglo, M. *et al*. Critical parameters in exfoliating graphite into graphene. *Physical Chemistry Chemical Physics* **15**, 4428-4435 (2013).

21. Zeqiri, B., Gelat, P. N., Hodnett, M. & Lee, N. D. A novel sensor for monitoring acoustic cavitation. Part I: Concept, theory, and prototype development. *Ultrasonics, Ferroelectrics, and Frequency Control, IEEE Transactions on* **50**, 1342-1350 (2003).

22. Backes, C. *et al*. Edge and confinement effects allow in situ measurement of size and thickness of liquid-exfoliated nanosheets. *Nature Communications* **5** (2014).

23. Backes, C. *et al*. Spectroscopic metrics allow in situ measurement of mean size and thickness of liquid-exfoliated few-layer graphene nanosheets. *Nanoscale* **8**, 4311-4323 (2016).

24. Paton, K. R. *et al*. Scalable production of large quantities of defect-free few-layer graphene by shear exfoliation in liquids. *Nature Materials* **13**, 624 (2014).

25. Pollard, A., Paton, K., Clifford, C. & Legge, E. Characterisation of the Structure of Graphene. Good Practice Guide No 145. (2017).
